# Cycles and circulation: a theme in the history of biology and medicine

**DOI:** 10.1007/s40656-021-00425-3

**Published:** 2021-07-12

**Authors:** Nick Hopwood, Staffan Müller-Wille, Janet Browne, Christiane Groeben, Shigehisa Kuriyama, Maaike van der Lugt, Guido Giglioni, Lynn K. Nyhart, Hans-Jörg Rheinberger, Ariane Dröscher, Warwick Anderson, Peder Anker, Mathias Grote, Lucy van de Wiel, the Fifteenth Ischia Summer School on the History of the Life Sciences

**Affiliations:** 1 Department of History and Philosophy of Science, University of Cambridge, Cambridge, UK; 2 Department of the History of Science, Harvard University, Cambridge, MA USA; 3 Naples, Italy; 4 Department of East Asian Languages and Civilizations, Harvard University, Cambridge, MA USA; 5 Université Paris-Saclay, UVSQ, DYPAC, Versailles, France; 6 Dipartimento di Studi Umanistici, Università di Macerata, Macerata, Italy; 7 Department of History, University of Wisconsin-Madison, Madison, WI USA; 8 Max Planck Institute for the History of Science, Berlin, Germany; 9 Bologna, Italy; 10 Department of History and the Charles Perkins Centre, University of Sydney, Sydney, Australia; 11 Gallatin School, New York University, New York, NY USA; 12 Institut für Geschichtswissenschaften, Humboldt-Universität zu Berlin, Berlin, Germany; 13 Department of Sociology, University of Cambridge, Cambridge, UK

**Keywords:** Canonical icons, Circles, Diagrams, Life cycles, Metaphors, Recycling

## Abstract

We invite systematic consideration of the metaphors of cycles and circulation as a long-term theme in the history of the life and environmental sciences and medicine. Ubiquitous in ancient religious and philosophical traditions, especially in representing the seasons and the motions of celestial bodies, circles once symbolized perfection. Over the centuries cyclic images in western medicine, natural philosophy, natural history and eventually biology gained independence from cosmology and theology and came to depend less on strictly circular forms. As potent ‘canonical icons’, cycles also interacted with representations of linear and irreversible change, including arrows, arcs, scales, series and trees, as in theories of the Earth and of evolution. In modern times life cycles and reproductive cycles have often been held to characterize life, in some cases especially female life, while human efforts selectively to foster and disrupt these cycles have harnessed their productivity in medicine and agriculture. But strong cyclic metaphors have continued to link physiology and climatology, medicine and economics, and biology and manufacturing, notably through the relations between land, food and population. From the grand nineteenth-century transformations of matter to systems ecology, the circulation of molecules through organic and inorganic compartments has posed the problem of maintaining identity in the face of flux and highlights the seductive ability of cyclic schemes to imply closure where no original state was in fact restored. More concerted attention to cycles and circulation will enrich analyses of the power of metaphors to naturalize understandings of life and their shaping by practical interests and political imaginations.

## Approaching cycles and circulation

Cycles run through the life and environmental sciences and medicine in the early twenty-first century. Organisms are understood as following life cycles, inherited sequences of stages through which they live, reproduce and adapt. Disrupting pest and pathogen life cycles plays key roles in agriculture, biomedicine and public health. Metabolism, development and inheritance are seen to depend on self-maintaining regulatory loops, including cell-to-cell and environmental signalling systems. Organisms are connected to each other, and to the air, soil, rocks and water, by biogeochemical cycles. The effects of human activities on the circulation not least of carbon, nitrogen and water threaten climate crisis, pollution and habitat loss. Omnipresent today, cycles are also among the oldest ways of framing human existence, and of thinking about life and death, health and disease, as well as daily and seasonal rhythms of regeneration. Here we consider such cyclic and circulatory metaphors as a long-term historical theme.

According to the *Oxford English dictionary* (*OED*), the English word *cycle*—derived from the Greek *κύκλος*, for circle or ring, and the Latin *cyclus*—refers to a process of recurrence, one that returns, or closes, upon itself, and thus defines both a period of time and a domain of repeated activities. Used in astronomy and for calendars, cycles have broadly indicated intervals “in which a certain round of events or phenomena is completed”, for example, the fixed series of days for which learned physicians had patients stick to a “course of remedies”.

These meanings overlap with those of *circulate* and *circulation*. In classical Latin, *circulus* and *circu(m)latio* were synonyms of *cyclus* in the astronomical sense of a circular course, as in the revolutions of the planets and the calendar. In the later medieval and early modern eras, *cyclus*, *circulatio* and the associated verbs took on additional meanings, including the cyclic transformation of matter from form to form and the motion and circulation of fluids. *Circulatio* was applied to the movement of blood from the sixteenth century and in alchemy referred to the refining of a liquid by continuous distillation in a closed vessel. According to the *OED*, these usages were expanded to “the movement of any thing in a ‘round’, not strictly circular” and the “transmission or passage of anything … from hand to hand”. In French, Littré’s dictionary reports a similar range of meanings for *cycle*, *circuler* and *circulation*, and further notes the Carnot cycle in thermodynamics, where cycles may be closed or open, reversible or irreversible. The Grimms tell us that German imported the word *Zirkulation* into alchemy in the early seventeenth century. In the eighteenth, along with its vernacular synonym *Kreislauf*, the term began to encompass planetary orbits, the circulation of the blood, and then (after long-established senses of *circulatio*) also that of such items as water and money. The German-speaking world has used *Zyklus* for life cycles since the early nineteenth century, and later adopted it for menstrual cycles.

In what follows, we sketch a history of metaphors of cycles and circulation in the life and environmental sciences and medicine, identifying patterns of continuity and change and paying special attention to their neglected visual dimensions, as a stimulus to further research. For the most part, historians have studied particular cycles in isolation. While making no claim to comprehensiveness, we are interested, over the long term, in similarities and dissimilarities across disciplines and among phenomena of different scales. Cycles have loomed large throughout human culture, including the mathematical, physical and social sciences, but we have sacrificed some breadth for a closer analysis of the ways in which living beings and life have been figured as cyclic and as involved in circulation.

Two previous attempts at synthesis are available in German. Engelbert Schramm’s history of ideas of the circulation of matter critiqued metaphorical assumptions that distracted from limitations in practices of recycling waste. Though ranging from antiquity to the present day, Schramm focused on the glorification of cycles in debates over sanitation and agriculture in and around industrial cities. The quasi-religious faith in the “eternal” cycling of “immortal” matter hid the inability of technical solutions to deliver on promises that they would close the cycle (Schramm, 1995). More generally, he argued, cycles gain ideological power by implying restoration but denoting processes that seldom return to their starting point in any strict sense.

Building on Schramm, and casting a wider net, Georg Toepfer’s 37-page entry on *Kreislauf* in his historical dictionary of “foundational concepts in biology” stressed the productivity of cycles as distinct from but related to circles. If the circle, as a figure without beginning or end, symbolized perfection and completeness, cycles represented dynamic processes of self-maintenance and reproduction held to be characteristic of living beings. Combining historical and systematic approaches, Toepfer proceeded by period and sub-discipline, and ventured a four-fold typology: material cycles, such as of the blood or chemical elements; developmental cycles, as in life cycles; causal cycles, in which several, otherwise independent bodies or processes stand in a relationship of mutual influence, as in regulatory loops; and cycles of dependency (*Bedingungskreisläufe*), for example, of the organs within an organism or the organisms in an ecosystem, in which no element would exist without the others. Here cycles encompass not just circulating bodies and matter and recurring events, but also periodic changes of state and even chains of causal processes that seem to close in on themselves (Toepfer, 2011).

Toepfer suggested that circles and cycles have fostered different kinds of visual engagement with organic processes from those offered by other geometrical figures. Put simply, if the line integrated animals, plants and minerals into the causal order of nature, as in the chain or ladder of being, then since around 1850 the tree has signified historical development with diversification, while the circle for the most part stood for the individuality of organisms as autonomous systems of interdependent elements. The net, with its non-hierarchical connections, became prominent in the later twentieth century (Toepfer, 2011, pp. 309–310). Maps and series have long represented organic relationships, too (Barsanti, 1992; Hopwood et al., 2010).

Following Stephen Jay Gould, we propose to treat the cycle as not only a set of concepts and metaphors, but also a canonical icon which epitomizes a major tradition of visualization and conceptualization (Gould, 1997). Yet there is more to it, as Toepfer’s analysis indicated. Like the much-studied trees of life (Hellström, 2019), cycles are powerful tools for thought. Whereas trees have helped their users to conceive of life in terms of both unitary origin and boundless differentiation, cyclic metaphors are more deeply ambiguous. The meanings of cycles vacillate between closedness, individuality and perfection, on the one hand, and openness, multiplicity and dispersion, on the other. As we shall argue, cyclic images began to gain some independence from cosmology and theology in later medieval and early modern European medicine, natural philosophy and natural history, and henceforth came to interact with representations of linear change, including arrows and arcs, scales, series and trees. The capacity of cyclic schemes to mediate between closed and open states, and to imply closure where the original state is not fully restored, explains their continuing attraction.

While agreeing with Gould that icons constrain as well as enable thought, we seek to go beyond the dichotomy that he presented for notions of history in *Time’s arrow, time’s cycle* (Gould, 1987). Rather, cycles are visual and rhetorical devices with distinct advantages and disadvantages in grasping the manifold phenomena of life. They may be flexibly deployed and can mesh with other representations. We aim to capture the diversity of often conflicting meanings in the dynamic history of this metaphor and to identify the practical, political and epistemic interests that have driven its use.

## Circles, arcs and arrows in microcosm and macrocosm

A long view makes visible themes that have shaped knowledge for millennia, even as the reasons for adopting them have varied and changed. The universalizing assumptions in Mircea Eliade’s *The myth of the eternal return* (1954) may be untenable (Chidester, 2018, pp. 47–57), but circles, wheels and cycles independently played large roles in all ancient systems, from the Egyptian solar calendar to the Buddhist wheel of life.

In the Chinese tradition, which we include by way of comparison, the symbol of yin-yang interaction that we know today evolved from circular, text-based diagrams that scholars devised in the eleventh century. But intuitions of cyclic order were already central to understanding the world in ancient China. Associated with the rhythmic circulation of *qi—*the vital breaths animating the world—around the cardinal directions, the four seasons framed all social life. Spring was truly springlike when *qi* streamed in from the east. In summer *qi* was supposed to blow from the south, in autumn from the west, and in winter from the north. All change in the world—from the *yang* expansiveness which arose in spring and peaked in summer, causing plants to flourish and insects to become active, to the *yin* retreat which began in autumn and ruled the winter, seeing trees go bare and animals hibernate—turned on the orderly circuit of transformative winds.

This cycle of *qi* lay at the heart of the *Huangdi neijing,* the classic of traditional Chinese medicine, and inspired two diverging approaches to health. One promoted the synchronization of human change and cosmic change with the aim of ensuring that the seasonal shifts inside the body matched those of the surrounding world. Loss of synchrony—say, an autumn- or winter-like pulse in summertime—signalled sickness. The second approach urged, on the contrary, the cultivation of a certain independence. Some insulation from the cosmos was vital, because the seasons were often erratic. Spring might come early; summer could be cool or autumn short; a balmy week could intrude into winter. Preserving health required protecting against such vagaries, which easily killed plants and animals. These contrasting approaches to nurturing life mirrored two different, but equally important aspects of traditional Chinese thought: the firm belief, on the one hand, in a world ruled by cyclic rhythms, and a keen awareness, on the other, of how chaos shadowed these cycles (Kuriyama, 1999).

In the classical Greek and Roman world—and this essay will henceforth be concerned primarily with the Mediterranean basin, North Atlantic and areas of European influence—cycles of generation and corruption, of life and death, and of the transformation of the elements likewise structured knowledge and many everyday activities. But the underlying cosmological assumptions were different. In Aristotle’s influential philosophy, heavenly cycles were perfect and unchanging, while the sublunary sphere emulated these movements in processes of coming into being and passing away. The revolutions of the celestial bodies were thought to shape the repetition of events on Earth, from the succession of the seasons, with their annual cycle of renewal, to the tides and women’s monthly bleeding (Freudenthal, 2010). Even before Aristotle, the Hippocratic treatise *Airs, waters, places* told physicians to consider first the seasons and their effects on bodies (Hippocrates, 1923, pp. 70–73, ch. 1–3). Human constitutions and fever cycles were determined by the motions of the stars, and subject to different complaints in different seasons, to which regimen had to be adapted.

As Hippocrates taught for living beings (Hippocrates, 1923, pp. 122–123, ch. 19), so historians reckoned that in political institutions, too, perpetual uniformity led to stagnation. Building on the cosmological premises of Herodotus in the fifth century BC, in the Hellenistic period Polybius elaborated the doctrine of anacyclosis, according to which dynasties went through cycles based on rule by the one, the few and the many, from monarchy then tyranny through aristocracy and oligarchy to democracy and demagoguery and back to monarchy (Herodotus, 1921, pp. 104–111, book 3, ch. 80–83; Schlosser, 2020, pp. 61–88; Walbank, 1964). In late fourteenth-century North Africa, Ibn Khaldun developed these ideas in his original reflections on cycles of consolidation and fragmentation (Ibn Khaldun, 1958). An alternative was the opinion, found in Plato and Aristotle, that natural disasters caused by the stars periodically destroyed cities and civilizations, which then rose again, so that history would repeat itself every 36,000 years. This longer cycle corresponded to the ‘great year’: the time it took for Sun, Moon and planets to return to their original positions. Medieval Arabic and Latin writers further developed—and criticized—this view (de Callataÿ, 1996).

Yet Aristotle at one point denied (though he elsewhere confirmed) that the generation of humans and beasts went in cycles; he deemed generation too directional, with offspring dependent on parents but not the other way round, for the process to count as truly cyclic (Toepfer, 2011, p. 316). Nor did the circular movement of the heavens map straightforwardly onto cycles of life. In the sublunary sphere life was governed by imperfection, resulting in cycles of generation and corruption that did not perform a full return. Many cycles, though depending for their representation on circles, therefore referred to processes that were not strictly circular. This foundational problem would trouble nearly all subsequent uses of cycles.

**Fig. 1:**
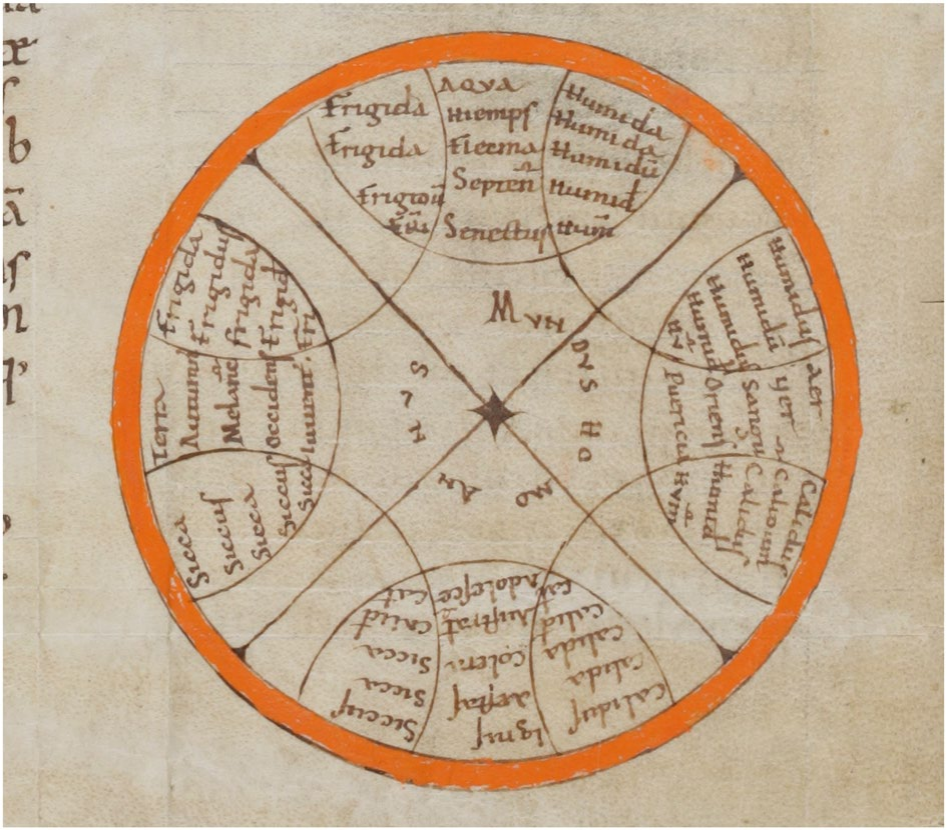
Mundus-Homo-Annus. This wheel-like diagram, from northern France in the ninth to eleventh centuries, associates the elements, seasons, humours, cardinal points and ages of man with pairs of primary qualities. The quadrant at the top reads: cold—water, winter, phlegm, north, old age—humid. Beda, *De temporum ratione*. MS Sankt Gallen, Stiftsbibliothek, 248, f. 148 (www.e-codices.ch)

The diagrams of ‘Mundus-Homo-Annus’ (world, man, year), which were used as memory devices in the middle ages, epitomize both the power of the circle to encourage cyclic thinking and the lack of necessary connection between the two. In wheel-shaped diagrams, of which some of the oldest accompany Isidore of Seville’s encyclopaedia, the circle denoted perfection (Obrist, 1996). The wheels make visible the logic, order and harmony of the created universe, the cyclicity of time and the analogical relations between the universe and the human body and life cycle (Fig. 1). But how did the wheels work? The sharing of one primary quality within each quadrant suggests the cyclic transmutation of the elements and the humours, while the addition of the seasons, the ages of man, the zodiac and, more rarely, the phases of the moon also invites dynamic readings (Schmitt, 2019, pp. 89, 109–113). Though there were three-, six- and seven-stage versions, an individual human life was most commonly represented as moving through four stages (childhood, adolescence, adulthood and old age), because these were easily integrated into a four-fold system with the seasons, the cardinal points, the elements, the primary qualities and the temperaments or humours (Burrow, 1988; Cochelin, 2013). Yet the life stages follow each other in a linear order, and other distinctions and connections within these diagrams are logical and not cyclic: fire, for instance, cannot turn into earth, but is opposed to it (Van der Lugt, 2011, pp. 283–284).

Cycles were similarly modified by the linear scale of nature and models of linear change over time. The scale, again representing logical rather than causal or temporal relations, was superimposed on individual life cycles. Though eternal, cyclic or steady-state views were the norm in cosmology, the Abrahamic faiths were all committed to directional histories. Calendars and almanacs converted these linear chronologies into the annual rounds of religious festivals (Rudwick, 2014, p. 29). Meanwhile, medieval scholars debated the argument, which came from Aristotle directly and via the Arabic tradition, that life is not a wheel-like cycle, in which every phase enjoys equal status, but rises like an arc to a plateau: “an age of standing still [and] of beauty”, in the words of the physician-philosopher Ibn Sina (Avicenna), writing in eleventh-century central Asia (quoted in Burrow, 1988, p. 23; Cochelin, 2013; Youngs, 2006, 2013). By the sixteenth century, this model of an arc of life also shaped visualizations, as depictions shifted from wheels to ascending and descending stairs. Privileging the middle stage, these popular images resonated with norms of intergenerational relations as peasant and bourgeois families became more important economic units (Ehmer, 1996; Schenda, 1983; Toepfer, 2011, p. 318).

From the thirteenth century, with the assimilation of ancient and Arabic medicine and natural philosophy into Latin scholarship, representations of the human life cycle had also started to lose their associations with cosmology. Scholars debated the physiology of ageing and ways to replenish the body’s ‘radical moisture’ (Crisciani & Ferrari, 2010; McVaugh, 1974). While efforts to prolong life were more secular, they still emulated the biblical patriarchs, who had lived for between 148 and a thousand years (Ziegler, 2018). Christ was crucified in his prime, and several church fathers argued that people who died as infants or at a great age would be resurrected as thirty-three-year-olds (Bynum, 1995, p. 122). The Christian promise of resurrection stressed restoration. The faithful thus proceeded through the ages of man or woman in the hope that individual death in this world would be followed by eternal life in the next.

Cycles were, then, not quite as widespread or dominant in the ancient and medieval worlds as they might seem, and this appears true of the Chinese tradition, too. Not all circles or wheels represented cycles, and these competed with and were modified by linear scales, arrows and arcs. The association between macrocosm and microcosm would be further questioned in early modern Europe, where cycles acquired lives of their own in a variety of domains.

## Cycles in bodies natural and politic

During the Renaissance and the Reformation, earthly cycles, with flexible geometries but defined points of return, diverged more clearly from the supposedly perfect circular orbits of heavenly bodies, for which it is arbitrary to specify any such points. Some metaphors of cycles stayed embedded in cosmological frameworks of analogies and affinities while others became relatively independent and attracted scholarly attention for their own, inherent regularity. As early modern natural philosophers engaged in the intellectual, social and political ferment of the age, they increasingly explored mundane, self-generating cycles as solutions to the challenges of reconciling change and restoration in sublunary bodies natural and politic.

In medicine, philosophy and politics, authors such as the diplomat Niccolò Machiavelli, statesman Francis Bacon and tutor Thomas Hobbes figured cycles as recurring patterns based on the interplay of natural uniformity and human changeability. Beginning in the fifteenth century, Renaissance humanists had already recovered Herodotus’ model of natural and historical recurrences (Olivieri, 2004). This helped reconnect the political debate to its cosmological roots as theorized in Greek and Roman antiquity. Machiavelli’s speculations on the life and death of commonwealths drew on the idea of the cycle of life (Machiavelli, 1998, p. 109). In politics, as in nature, the struggle for survival and the desire to persist drove human affairs, which were ruled by cycles of rise and fall, progress and downturn, growth and decay. Machiavelli called this motion “freedom” or “free life” (Giglioni, 2021; Machiavelli, 1998, pp. 118–124). For Bacon, an attentive reader of Machiavelli, the cycle of life in nature and politics similarly oscillated between the desire to escape immobility (“motion of freedom”) and the desire to shun disintegration (“motion of repose”; Bacon, 2004, pp. 410–412). Hobbes reinterpreted Machiavelli’s and Bacon’s ideas about natural and social motion in terms of his mechanical philosophy. When an object met the senses, perception plus the vital motion of the blood, “perpetually circulating”, let the body respond with appetite or aversion. In animal motion, wrote Hobbes, “this is the very first endeavour” (Hobbes, 1994, p. 226).

Machiavelli, Bacon and Hobbes all approached cycles in similar ways. Life consisted in forms of primal reactivity, and the reactions of natural bodies explained those of more complex organizations, including bodies politic. Whether this original reactivity was a natural desire or a reflex resulting from the mechanical arrangement of the parts, larger conglomerations of individuals could be understood by assuming a basic ability to respond to opportunities and threats. Evolving patterns of *conatus*, *conservatio* and *crisis*—that is, appetite, self-preservation and tipping point—thus shed light on the characteristic cycles of bodies politic with their fluctuations involving appetite for life and freedom, but also fear of death, dread of change and desire to rest. In the background, one can perhaps discern the attempts of emerging states to harness circulation to bolster their power and wealth (Foucault, 2007, pp. 1–27).

By contrast, William Harvey’s discovery of the circulation of the blood, to which Hobbes referred, illustrates in its more traditional Aristotelianism the continuing symbolic importance of circles, and the enduring links between microcosm and macrocosm in the sixteenth and seventeenth centuries. Andrea Cesalpino had written in 1571 of the blood as undergoing a rhythmically repeated *circulatio*, in the alchemical sense of continuous distillation. In 1628, Harvey proposed that it rather moved in a *circuitus* through the whole body. But in defending his views, he brought *circulatio* back and his followers would go on framing the process as distillation. The medical historian Walter Pagel called Harvey, like Cesalpino an Aristotelian physician, a “philosopher of circles” (Pagel, 1957). The heart was the equivalent of the Sun in the microcosm—and the king in the body politic—and if blood circulation could never match the divine order of the celestial orbits, it did its best to imitate them. It also became a paradigm, among Harvey’s contemporaries and in the next generation, for the flow of everything from matter to money and inspired a theory of sap circulation in plants, which though never mainstream, found adherents until the mid-nineteenth century (Delaporte, 1982, pp. 31–89; Kirschner, 2002).

Aristotle had compared the veins of the Earth and the body, observing that too much water flows in rivers to be distributed from an underground reservoir. Instead, it must circulate. Harvey developed this quantitative argument in reasoning that the rate of blood flow through the heart is too great for supply from the vessels (Gregory, 2001). When he went on to explore animal generation, Harvey employed similar links between microcosm and macrocosm. Where the soon-orthodox view, preformation, “implies a once-and-for-all process which unwinds and must eventually come to an end”, Harvey’s favoured theory, epigenesis, “is a cyclic process which can continue forever” (Pagel, 1957, p. 155). While the earliest structure appearing in the embryo, the ‘pulsating point’, seemed to form the fresh beginning of each individual life, the development and succession of individual beings within a species, Harvey suggested, had to be seen as embedded in the seasonal cycles generated by the circling of the Sun (Müller-Wille, 2007). The circle as the shape of perfection may have tamed anxieties about the disorder represented, for example, by the possibility of the spontaneous generation of life and the threat this posed to patriarchal authority (Keller, 2007). As in Galilean mechanics, cyclic metaphors gained prominence in attempts to see celestial perfection in the sublunary sphere.

**Fig. 2:**
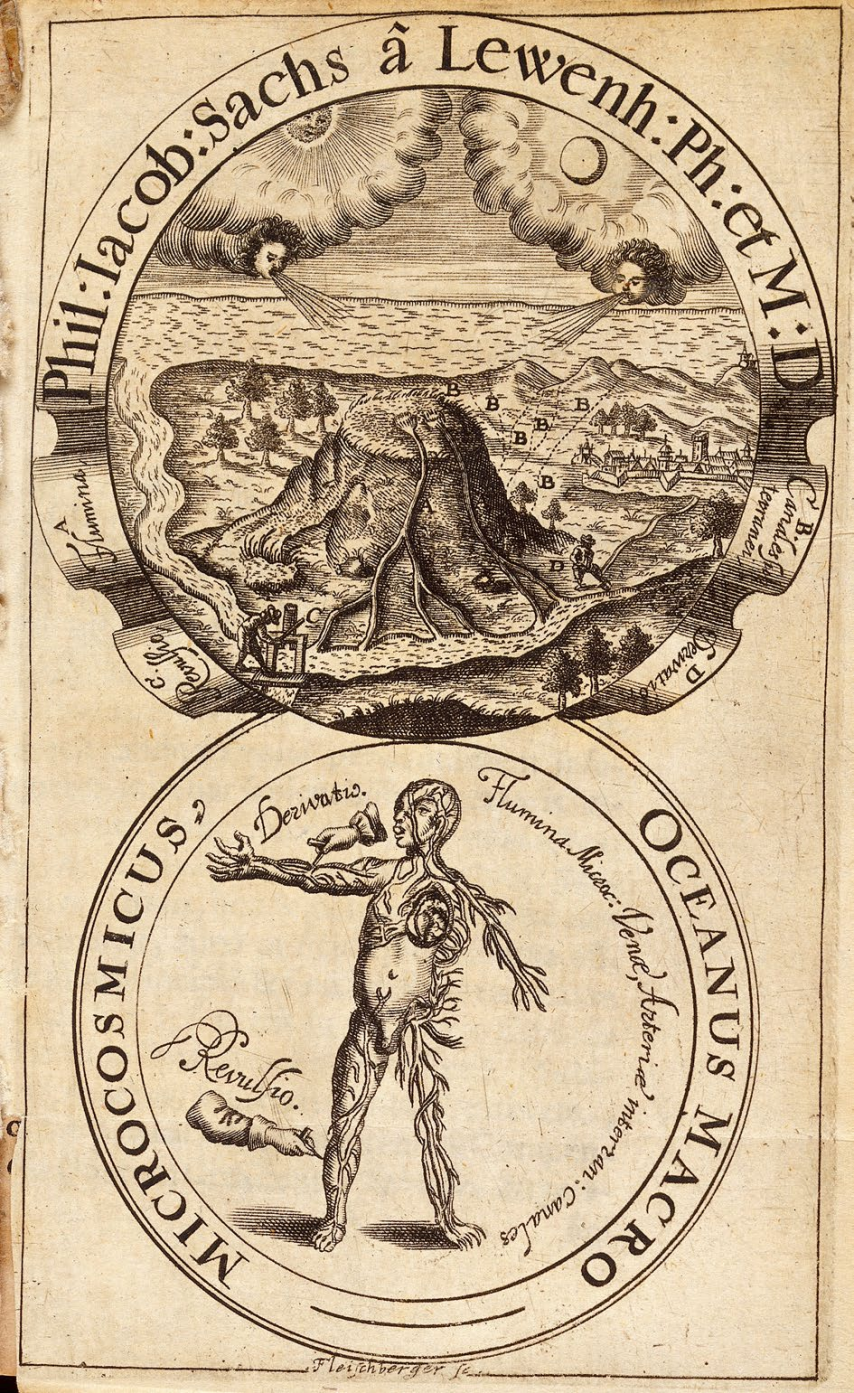
The analogy between meteorology and physiology in the frontispiece to Philipp Jakob Sachs von Lewenhaimb’s *Oceanus macro-microcosmicus*. The two engravings, fittingly placed in circular frames, show how the Sun and Moon propel water through a circular system of rivers and “subterranean canals”, just like the heart drives blood through arteries and veins. Sachs von Lewenhaimb (1664). Wellcome Library, London

The water cycle, in something like its modern form, was put together in the late seventeenth century. Natural theologians, notably John Ray in *The wisdom of God manifested in the works of the creation* (1691), combined the meteorological water–evaporation–rain cycle with the hydrogeological flow of water through the rivers into the seas. This providential arrangement, which Ray did not call circulation or a cycle, served to provide clean water for human use. Previously, it had been common to postulate that water returned from the sea underground as, for example, in the rare picture of the macrocosm–microcosm analogy between meteorology and physiology that is the frontispiece to a 1664 book by the physician and naturalist Philipp Jakob Sachs von Lewenhaimb (Fig. 2). Later writers secularized the water cycle but kept much of the teleology and assumptions about purification (Schramm, 1995, pp. 95–116).

Early modern European knowledge was, then, much structured by cycles, with lively traffic between domains of knowledge. But although some writers appealed through analogies between micro- and macrocosm to the very architecture of the universe, others merely found the same drives at work in bodies natural and politic. Circles might symbolize perfection, but cycles no longer had to follow fixed circular paths (and planetary orbits themselves came to be understood as ellipses). This greater autonomy from cosmology set the stage for change, and meant that cycles could be treated as objects of inquiry in their own right as well as linked across fields. The next three sections explore the implications for conceptions of history, approaches to generation and reproduction, and analyses of the transformations of matter.

## Time’s cycle and the arrow of history

Cyclicity often serves as a foil to progressive change, yet cycles have their own specific histories, and these have often been integrated with linear models since antiquity. Late seventeenth- and eighteenth-century ‘Theories of the Earth’ exhibit extreme confrontations and elaborate conciliations between cycles and arrows. Stimulated by René Descartes’ conjectural physics of an irreversibly cooling planet, and emulating the ambition of Isaac Newton’s celestial mechanics, their authors variously combined cycles and directional change in all-encompassing causal explanations of the Earth’s physical features, past, present and future. In the early nineteenth century, however, cycles were marginalized as the conception of a linear, finite and contingent history was definitively transferred from the social to the natural world (Gould, 1987; Rudwick, 2005, 2008, 2014).

**Fig. 3:**
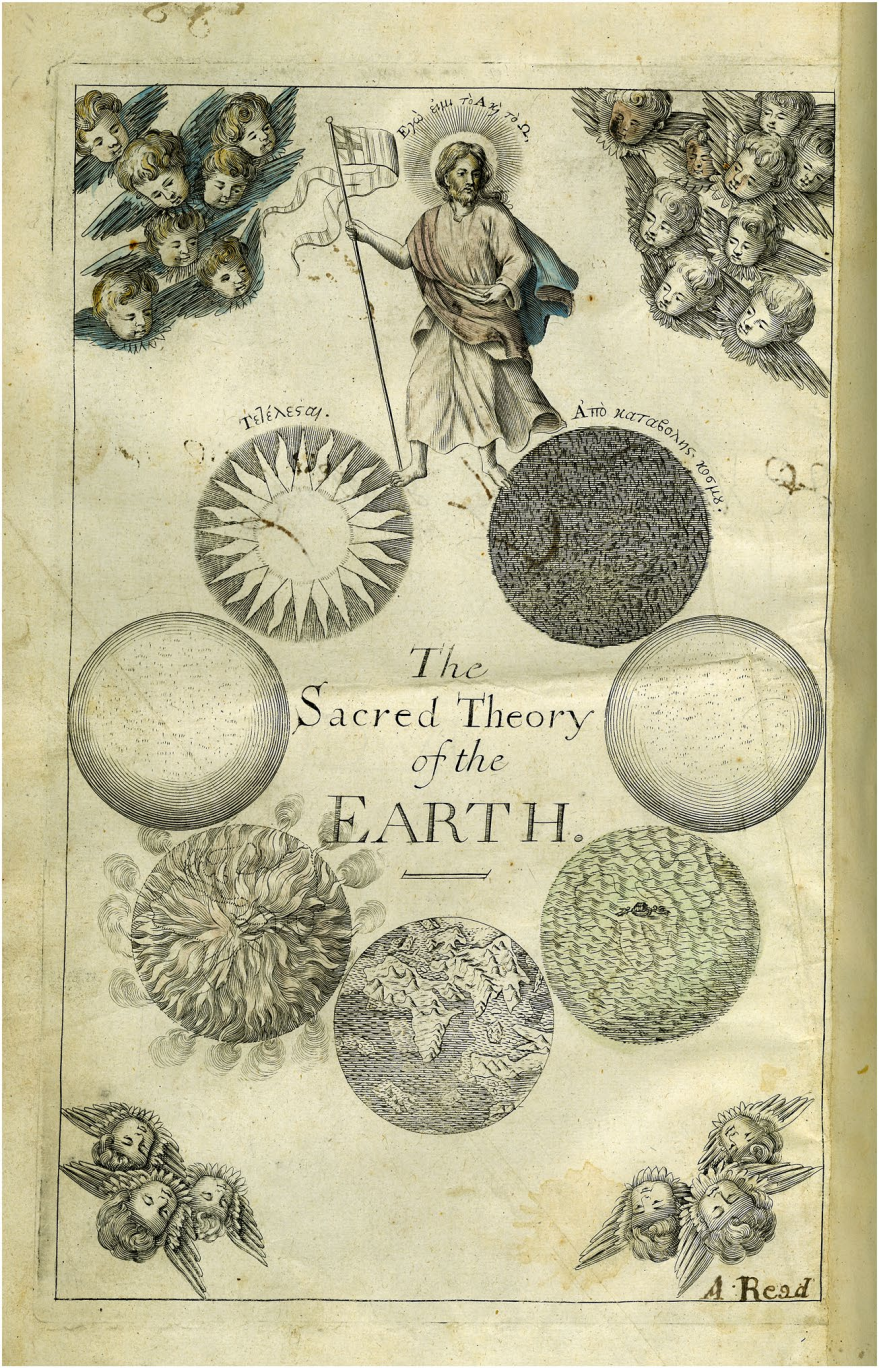
Frontispiece to the English translation of Thomas Burnet’s *Sacred theory of the Earth*. The coloured engraving shows the stages (clockwise from top right): Earth in chaos; the antediluvian Earth as perfect and featureless; Earth in the Flood, with the ark; the imperfect Earth we know, with its jagged continents and oceans; Earth in conflagration; Earth perfect again; Earth as a star. With a clear beginning and a clear end, the sequence is symmetrical with respect to past and future; angels look on from the eternal heavens. Andrew Read was a previous owner. Burnet (1684). University of Otago Library

Christian theology required an Earth that was created and ultimately destroyed, a form of directional history that contradicted Aristotle’s eternal world. The English cleric Thomas Burnet’s *Sacred theory* (first published in Latin in 1681 and 1689) integrated evidence from nature and scripture in a great circle of creation, decay and restoration. The frontispiece to his book shows the seven stages of history since the creation, arranged in a circle, with Christ the redeemer (“I am the Alpha and the Omega”) astride the initial chaos and the end, the Earth as a star (Fig. 3). Burnet’s scheme displayed, on the one hand, a linear narrative (but of return) arranged in a circle and, on the other, a cycle that repeated but with crucial differences: of destruction (by water) following perfection in the past and of perfection after destruction (by fire) in the future (Gould, 1987, pp. 20–51; Rudwick, 2014, pp. 58–60).

By contrast, the director of the French king’s garden, George-Louis Leclerc, comte de Buffon, began his multivolume natural history in 1749 with an essay that sparked intense controversy with theologians at the Sorbonne. It presented the modern world in constant flux, yet always ultimately the same because cyclic local alterations cancelled each other out. Thirty years later, Buffon abandoned this steady-state view; he drew on Pierre-Simon Laplace’s theory of cosmic origins to argue that the Earth formed naturally from a red-hot mass of rotating gas which solidified as it cooled. A sequence of changes led through seven epochs that broadly matched the biblical story. Though directional, these physical alterations and the consequent transformations in flora and fauna were predictable: they approached ever closer to the present day. Despite its definite beginning and end, Buffon’s was not a contingent history of the Earth or of life (Rheinberger, 1990; Rudwick, 2005, pp. 139–150).

Other Enlightenment savants joined the early Buffon in proposing that nature ran in perpetual cycles. James Hutton of Edinburgh, who participated in debates over the water cycle and other cycles of matter, was impressed by the tirelessly cycling steam engines his friends were inventing. First presented in 1785, his theory of the Earth is more cyclic than any other before or since. Inspired by a deist vision in which God had created the Earth for human beings, Hutton aimed above all to solve the problem of maintaining the fertility of the soil, itself formed from the breakdown of bedrock, in the face of its continual washing away into rivers and then the sea. He sought “a reproductive operation, by which a ruined constitution may be again repaired, and a duration or stability thus procured to the machine, considered as a world sustaining plants and animals” and so permanently fit for human habitation. Hutton found that operation in a scheme whereby rocks were laid down on the sea floor and then lifted up by crustal expansion to generate new land and let the process start all over again. Like the oscillations of a Newcomen engine, and the planets orbiting the sun, the world-machine would run on with “no vestige of a beginning,—no prospect of an end” (Hutton, 1788, pp. 216, 304; Rudwick, 2005, pp. 160–172).

In the early nineteenth century, at the Museum of Natural History in Paris, Jean-Baptiste Lamarck also offered a cyclic view of living processes and the environment, a ‘transformist’ zoological philosophy in which spontaneous generations continually initiated series of living forms of increasing complexity, some of which arrived at human beings. Lamarck’s series of ascending organisms were more or less linear but their death and decay formed the soil and rocks in an eternal circulation of matter (Corsi, 2011; Giglioni, 2013).

By this time, however, most writers agreed that Earth history was neither eternal nor cyclic. To them, the geological record suggested that the Earth had moved through several stages from its first beginnings, each stage approaching ever closer to the present. Irreversible change was evidenced most conclusively through the extinctions documented by the comparative anatomist Georges Cuvier. For Earth theorists, who had already worked with large amounts of time, the decisive innovation was the transfer of the Judaeo-Christian tradition of finite, linear and above all contingent history from the human to the natural world. Savants now laboured as ‘natural antiquaries’ to interpret nature’s archives, monuments and coins. Time became not a parameter but an operator, not a measure of the flowing water, but the river itself (Rudwick, 2014).

**Fig. 4:**
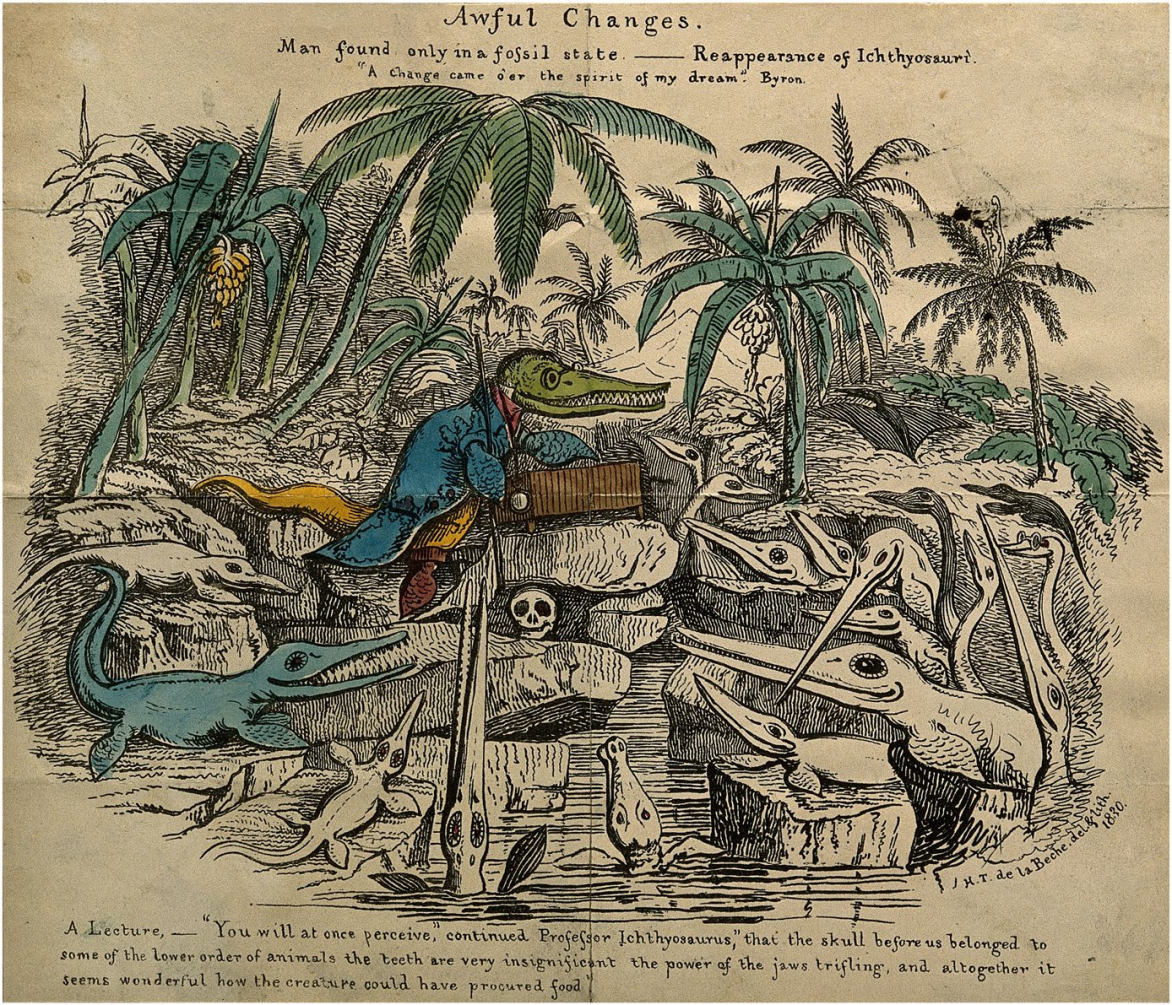
“Awful changes”. Caricature of Charles Lyell drawn and lithographed by Henry De la Beche in 1830, and partly coloured in watercolour. Wellcome Library, London

From a deep abhorrence of Lamarck’s transformism the Scottish barrister Charles Lyell challenged the consensus (Rudwick, 2008, pp. 297–330). Lyell asserted that directional changes were only apparent, not real. Seemingly progressive movement could be reversed, he showed, as by the small shifts that had elevated the Temple of Serapis, at Pozzuoli north of Naples, by several metres (Ciancio, 2009). Working to obliterate Lamarck’s system of linear development towards humans, Lyell undermined the assumption of advance from lower to higher organisms. While he did not advocate cycles in the history of life, he left open that possibility should the same conditions return. In *Principles of geology* (1830) he speculated that a changing Earth could foster successive creations and extinctions such that “[t]he huge iguanodon might reappear in the woods, and the ichthyosaur in the sea” (quoted in Rudwick, 1975, p. 558). Fellow geologist Henry De la Beche targeted this vision in a lithograph that imagined a Professor Ichthyosaurus lecturing on a human skull as a relic of a “lower order of animals” (Fig. 4). This satire lampooned cycles as a reactionary pushback against progressive views of life.

Other non-linear systems used circles explicitly, most famously the quinarian classification devised by the entomologist William Sharp Macleay in 1819 and discussed for the next twenty years. Based on groups of five circles at each taxonomic level, Macleay tried, while avoiding too simple a series, to represent more continuity than Cuvier’s four ‘embranchements’ had allowed. Macleay rejected transformism and the possibility of transitions from one group to the next, but touching circles were intended to show similarities between groups, such as the affinities of cephalopods with molluscs and vertebrates (Novick, 2016; see also Van Putten, 2019 on Paul Dietrich Giseke).

In this Age of Revolutions, debates over change in the natural world were bound up with social and political transformation and with shifting connotations of the word ‘revolution’ (Latin, *revolutio*). Having once designated the closed, circular movements of heavenly bodies, this term came to mean also a radical and irreversible transformation in political order and world view (Cohen, 1985). Distinct from the early modern uses of ‘instauration’, ‘innovation’, ‘rebellion’ or ‘schism’, ‘revolution’ implied inexorable progress to a point of no return. Various cyclic models still competed with and were incorporated into progressionist accounts of historical change, but these became dominant during the nineteenth century. By the mid-twentieth, progressionism was under sustained attack in the name of not cycles, but a more thorough-going contingency (Bowler, 1989, 2021). Yet cycles continued to figure centrally in connecting evolution with the fundamental processes of generation and reproduction.

## Life cycles and reproductive cycles, representation and control

The stages of an individual life might be represented in linear fashion from birth to death, but ‘reproduction’, newly recognized from the mid-eighteenth century as a shared power of organisms to beget their like and maintain the species over time, complicated this sequence. The term gained currency in projects to replenish natural resources, acclimatize exotic species and breed better plants, beasts and humans in Europe and its colonies. Meanwhile, the older word ‘generation’ took off in the sense of a cohort of the same age or at the same distance from an ancestor (Hopwood, 2018a; Parnes et al., 2008). This would intersect with early nineteenth-century physiologists’ beginning to write of life as typified by cyclic processes (Wellmann, 2017, pp. 135–154). The most distinctive of these—life cycles and reproductive cycles—were much investigated in the following decades. The *Encyclopaedia Britannica* entry on “Biology” cited the “tendency to undergo cyclical changes” as a “distinctive propert[y] of living matter”; “the continuation of the kind being secured by the detachment of portions which tend to run through the same cycle of forms as the parent” (Thiselton-Dyer, 1878, p. 679). Across the twentieth century, technological intervention would increasingly target such cycles.

From the 1820s researchers began to wrestle in earnest with the problem of life cycles as links between generations. Most research on animal generation having been done on vertebrates, the great variety of invertebrates presented new challenges, and many researchers looked for commonalities with plants as well. A heated debate among anatomists and physiologists, zoologists and botanists over ‘alternation of generations’ focused attention on the cyclic features of the life-course. The word *Lebenszyklus* became standard then—it had been introduced into German a few decades before, alongside ‘cycle of generation’ and ‘cycle of development’ (*Zeugungskreis* and *Entwicklungskreis*)—and ‘life cycle’ definitively entered English through the writings of the comparative anatomist Richard Owen.

**Fig. 5:**
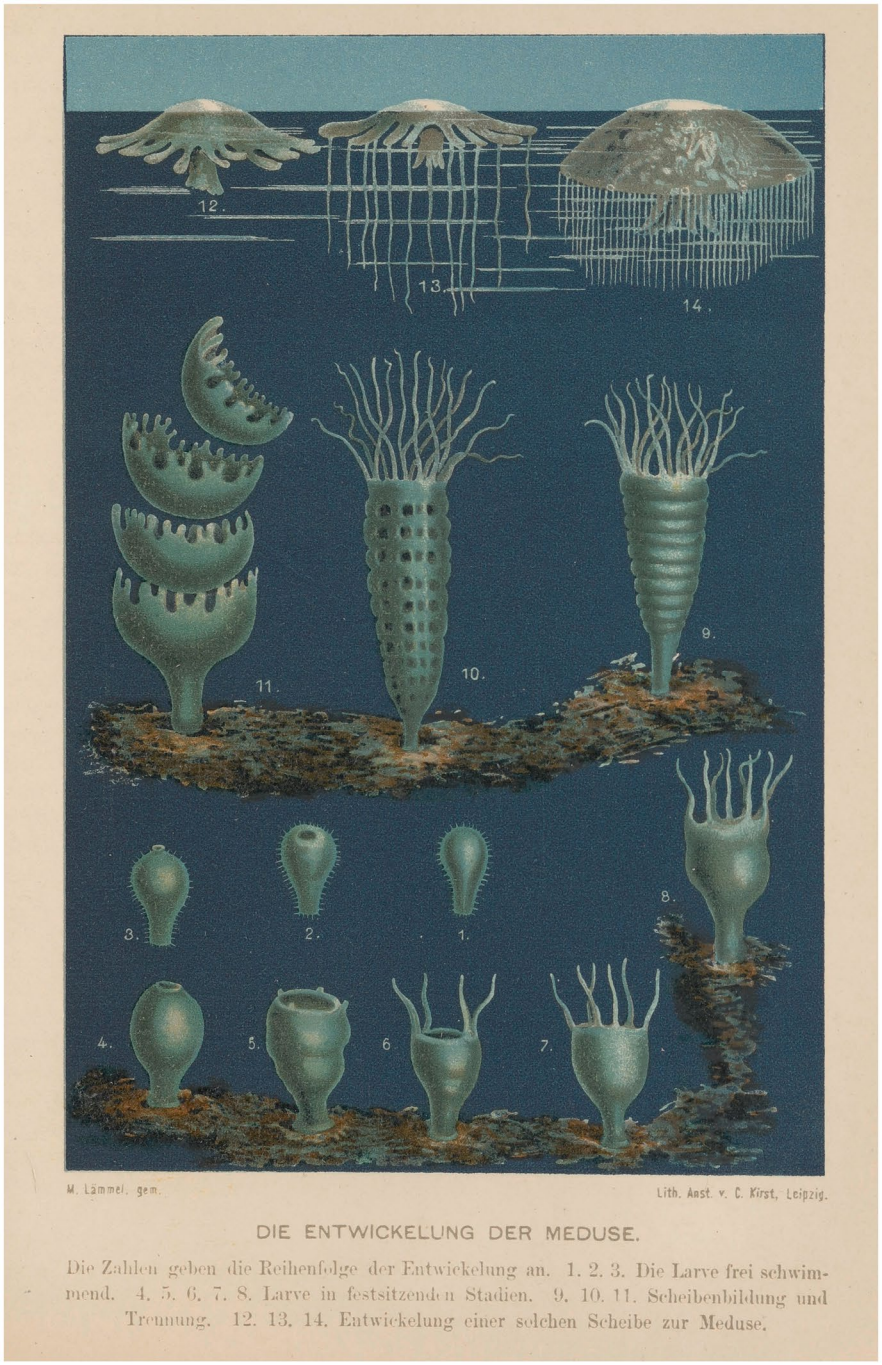
Development of a medusa (jellyfish) in botanist Matthias Jacob Schleiden’s *Das Meer* (The ocean), a successful book for a wide, educated audience. 1–3, free larvae; 4–7, sessile larvae; 8–11, asexual division and separation into disks; 12–14, development of disk into adult medusa. Development is figured as not a circle but a sinuous series that places each stage on the floor and then the surface of the sea as (in this new version for the posthumous third edition) it snakes largely up (rather than down) the page. Colour-printed steel engraving from Schleiden (1888, unnumbered plate facing p. 382). Universitäts- und Landesbibliothek Darmstadt

Insect metamorphosis had fascinated naturalists since the late seventeenth century (Ogilvie, 2014; Wellmann, 2008), but in the early nineteenth it was discovered that a species could also appear in two different forms such that an individual resembled its grandmother and granddaughters, but not its mother or daughters (Parnes, 2007). In 1842 the Danish naturalist Japetus Steenstrup generalized this break with assumptions based on vertebrates. Animals that had been supposed to be distinct species were reclassified as alternating forms of a single one. Concurrently, the discovery that these different forms reproduced differently—asexually or sexually—undermined linear views of maturation and reproduction (Fig. 5).

Naturalists debated whether to think in terms of different generations or the stages of one individual. For Owen, in 1849, “a species is represented by a series of individuals of different powers and forms succeeding each other in a cycle”, so a life cycle might take more than an individual lifetime to complete (quoted in Nyhart & Lidgard, 2017, p. 137). Thomas Henry Huxley countered that an individual was defined not by separate existence, but as the sum total of the development of a single ovum, and hence included what would otherwise appear as separate individuals. For the leading Darwinist Ernst Haeckel, similarly, “the product of the egg” (*Eiprodukt*) in sexually reproducing animals represented “the closed cycle of successive conditions of form …, which are constantly repeated within the species in rhythmic alternation from the egg of the mother to the egg of the child” (Haeckel, 1866, pp. 28–29). The closed cycle was the essential point. In botany, cycles were already familiar from plants’ annual dying back and renewal, and daily floral openings and refurlings synchronized with daybreak and nightfall; botanists by convention illustrated all stages in the life cycle, from the seed to the mature plant, on the same plate (Nickelsen, 2006). Researchers further emphasized cyclicity in two ways: by generalizing the alternation of generations across the (then) two kingdoms of life, and by shifting attention from form to processes of generation and development. Individuals and life cycles ceased to map onto each other one-to-one (Nyhart & Lidgard, 2011, 2017).

Attention to life cycles presented a problem, however: how to reconcile the cyclic patterns with the notion, to which these researchers were also committed, of development as progress to a higher state? The German botanist Alexander Braun sought an answer in the idea of rejuvenescence (*Verjüngung*) or renewal. According to Braun, cycles of maturation, destruction and renewal linked all scales of living nature, from the cell to the planet, in a nested dynamic of development. Driven by a higher purpose inherent to life, the cycle at each level contributed to development at the next. “Mother cells” matured and dissolved into their “daughter cells”. In simple algae (Braun’s specialty), this cellular dissolution yielded separate individuals—and thus new cell “generations”. In multicellular organisms the cell cycles collectively produced the plant or animal. Over geological time, through incremental cycles of destruction and renewal, species developed and then disappeared in “a single history of development, and not a series of separate and independent creations” (Braun, 1851, p. 11). Destruction and subsequent rejuvenation at each level produced development at the next higher one, with the ultimate purpose of realizing the “organism of nature” as a whole, culminating in the appearance of humans. The revolutions of 1848–49 had disrupted the work of the conservative Braun; his developmentalism showed how cycles of destruction could be incorporated into the purposive order of natural law.

In many nineteenth-century theories, change occurred via the alteration of life cycles through time. Influential authors explored evolution resulting from malformations that interrupted or redirected cycles of embryonic development; Robert Chambers’s system of transmutation is an example. Even Charles Darwin’s and Alfred Russel Wallace’s natural selection worked on random variations produced amidst endless repetitions of developmental cycles. Within the evolutionist frameworks that predominated later in the century, the doctrine that ontogeny recapitulates phylogeny promised repeated return, albeit in condensed and ‘falsified’ forms that allowed for either progress or degeneration. Some theorized that retardation or suppression of stages in a life cycle could lead to new forms, such as might explain the Mexican axolotls sent to Paris in 1864. In the early twentieth century, Louis Bolk, an anatomist in Amsterdam, suggested that humans arose by fetalization of chimpanzees (Churchill, 2007; Gould, 1977; Reiß, 2020). Connecting changes within and between generations to long-term transformations remained taxing throughout the twentieth century (Laubichler & Maienschein, 2007). Few emulated the Princeton biologist John Tyler Bonner in making the life cycle the central unit of evolution, heredity and development (Bonner, 1965), but it has been argued that genetics and evolutionary biology endowed reproductive cycles with the capacity to produce change (Griesemer, 2000).

**Fig. 6:**
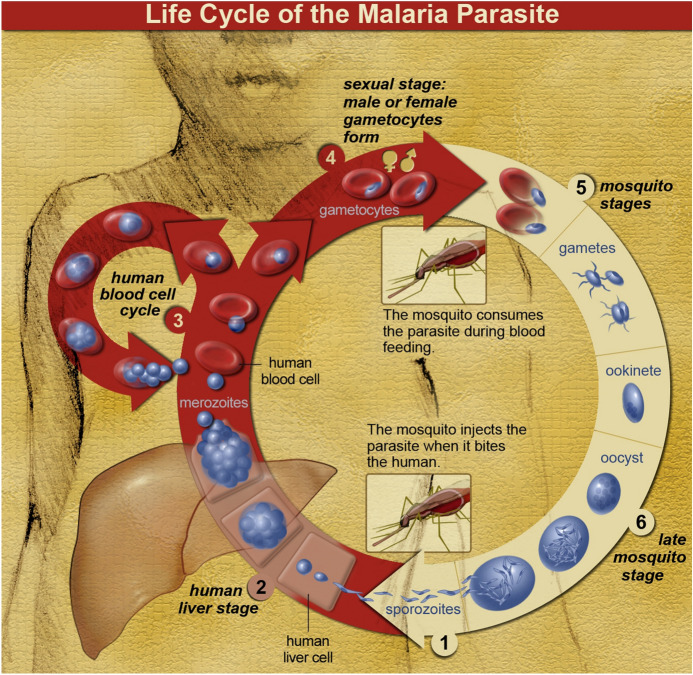
Diagram of the life cycle of the malaria parasite by the U.S. National Institute of Allergy and Infectious Diseases. Developmental stages of *Plasmodium* are arranged in a circle, the prominent numbers and arrows signalling direction and inviting discussion of intervention. The cycle begins at the bottom, when the mosquito bites the (dark-skinned, not obviously gendered) human host, and proceeds clockwise through liver (pink-brown) and blood (red) to vector stages (yellow) and back to host. Though return to the same human is shown, a member of the same species is meant. A smaller circle represents the proliferation of the parasite in human red blood cells. NIAID, 2007

On shorter timescales, representing and intervening in cycles have gone together in the life and medical sciences since the later nineteenth century. Some of the first and most prominent interventions went after parasites and microorganisms. Around 1900, life cycles of protozoan parasites through geographically restricted insect vectors and human hosts contributed to distinguishing the specialty of tropical medicine from ‘cosmopolitan’ bacteriology (Worboys, 1996). Targeting parasites via their vectors—such as attempting to eliminate malaria by eradicating mosquitoes—offered new points of intervention. Yet tropical hygiene largely ignored the living conditions of colonized people. Artists displayed mastery by ordering the various forms of a species on the page and adding arrows to show the direction of change, but diagrams of parasite life cycles tend even today to marginalize the life histories and social worlds of the humans involved (Fig. 6).

More generally, the collaboration of science and medicine in controlling human and animal reproduction has increasingly focused on life cycles and reproductive cycles. Women’s monthly bleeding, long discussed as a kind of ebb and flow, was in the nineteenth century pathologized as a periodic illness that disqualified middle-class girls from training for the professions (Rowold, 2010; Smith-Rosenberg, 1973). The bourgeois ideology of separate spheres assigned women to the home and to a cyclic domestic time that was rhetorically opposed to the linear, historical time of political events and to the working time of industrial capitalism (E. Thompson, 1967). Yet, irrespective of sex, all animals and plants and some microorganisms have come to be understood as subject not just to seasonal, but also to circadian (approximately daily) rhythms. Mid-twentieth-century researchers into such ‘biological clocks’ organized themselves in cross-disciplinary societies devoted to ‘chronobiology’ (Ahlheim, 2018, pp. 465–537; Cambrosio & Keating, 1983; Shackelford, 2013; on sleep: Kroker, 2007). By this time, the expectation that social arrangements should follow from women’s monthly periodicity was challenged by growing female participation in public life and in industry (Freidenfelds, 2009, pp. 74–119), and by reproductive technologies that could overcome (though also reinforce) assumptions of a natural cyclicity.

**Fig. 7:**
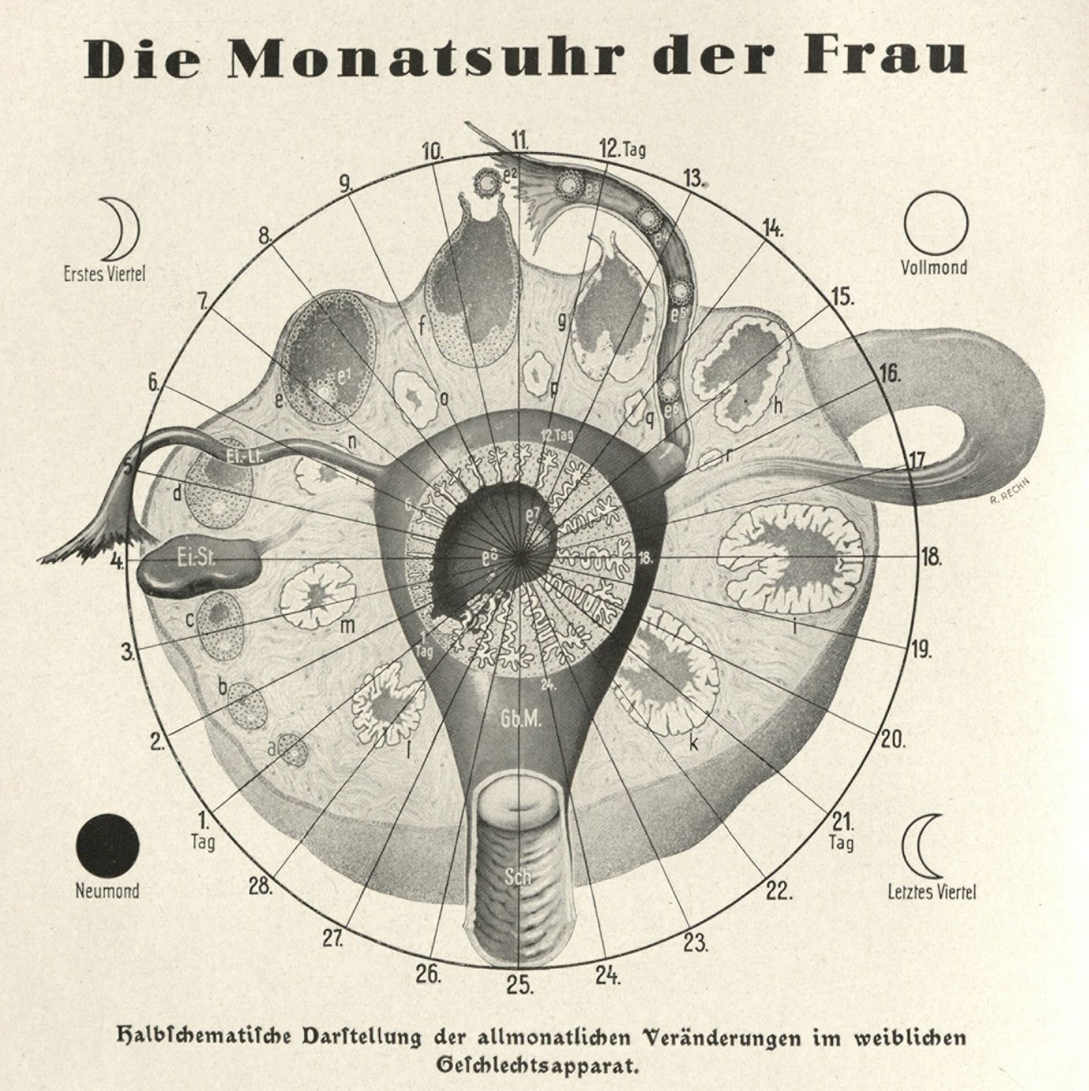
“Woman’s monthly clock” in physician-writer Fritz Kahn’s modernist classic, *Das Leben des Menschen* (Human life). Kahn’s artist, Roman Rechn, half-schematically mapped the days from 1 to 28 onto “the changes in the female sexual apparatus that happen every month”: in the disproportionately enlarged left ovary that fills most of the space, (a–r) the development of the ovum, ovulation (roughly on day 10) and development of the corpus luteum (the small right ovary is labelled Ei.-St.); (e^1^–e^7^) the egg developing into an embryo in ovary, fallopian tube (Ei.-Lt.) and uterus (Gb.M.) if fertilized, or (e^8^) degenerating if not; and, under the influence of the ovary, the thickening of the mucosal lining of the uterus until day 24 when, in the absence of an implanted embryo, the lining degenerates to produce the monthly bleed. Kahn explained that, although four phases of the moon were shown, there was no temporal or causal connection to them. Kahn (1931, plate XVI), courtesy of Michael Sappol

In the 1920s and 1930s anatomical and physiological research had reframed women’s reproductive function in terms of menstrual cycles, the higher-primate versions of other mammals’ oestrous cycles. Menstruation was reinterpreted histologically and biochemically as the breakdown of the lining of the uterus, driven by hormonal changes in an ovarian follicle; its timing in relation to ovulation was also finally agreed upon. Some forms of contraception observed this cycle and others intervened in it. Women had annotated calendars for centuries, but now the gynaecologists Hermann Knaus and Kyusaku Ogino promoted the rhythm method as a new practice of calendar marking (Kassell, 2018; Schlünder, 2005, 2018) (Fig. 7). Around the same time, female sex hormones were marketed as precise regulators of the menstrual cycle and, from 1960, as contraceptives (Olszynko-Gryn, 2018; Oudshoorn, 1994). In animal breeding the success of artificial insemination and embryo transfer also depended on the evaluation and manipulation of cycles (Hopwood, 2018b).

Though women’s reproductive cycles are still most often manipulated to prevent pregnancy, infertility rose up the agenda in the 1970s. After the birth of a baby following in vitro fertilization (IVF) in 1978, entrepreneurs turned reproductive failure into a billion-dollar industry that has made millions of families (and disappointed many more) (Hopwood, 2018b). Going through IVF involves turning menstrual cycles into treatment cycles, coordinating these with bureaucratic schedules, accommodating them within domestic routines and reporting per-cycle success rates to regulators (C. Thompson, 2005). While eugenics focused on preventing the births of those with disabilities, the poor and the non-white, reproductive biomedicine has targeted affluent and at first also primarily heterosexual, married and white consumers—a group that is still disproportionately represented in fertility clinics today. In the prevailing view of women’s age-related infertility, each ovulatory cycle implies another tick of the ‘biological clock’, that is, the linear ageing of egg cells. Since 2012, this framing of fertility decline has justified offering infertility treatment to fertile people via egg freezing. Fertility clinics increasingly market the possibility of extending a woman’s potentially fertile years (Van de Wiel, 2020).

More generally, developmental cycles came by the twentieth century to be recognized as responsible for the reproduction not just of gametes, but of all cells. ‘Cell cycle’, which had meant various things, dominantly referred to the succession of changes between two cell divisions (Crvčková, 2018). Chemical inhibitors aided their analysis and some of these have been deployed as agents of mutagenesis and chemotherapy. Heated debate over claims to ‘immortality’ and its limits—the number of times a cell population can cycle or divide—accompanied the invention of culture systems for vertebrate tissues (Landecker, 2007). ‘Stem cells’ were distinguished in embryology and haematology by their both dividing to produce cells that differentiated through linear development and participating in cycles of self-renewal. The idea that lingering embryonic cells developed into cancers once drove much research; recently, the manipulation of human embryonic stem cells has opened up the prospect of regenerative medicine (Dröscher, 2012; Maehle, 2011).

A great deal of academic biology, biomedicine, and also agriculture, can thus be grasped as working with life cycles and cycles of development and reproduction. Valuing, restoring and extending those of interest while rejecting, excluding and inhibiting the rest has been central to harnessing the proliferative productivity of life in fields and lakes, factories, laboratories and clinics. Yet because cycles disrupt the capacity of organisms to produce without regard to seasonal, monthly or diurnal change, efficiency has often demanded that cycles be damped down. Historians might pay more systematic attention to interventions that have halted or smoothed cycles for the pursuit of health as well as profit. These range from year-round crops and model organisms that breed in any season to treating bipolar disorder (alternating periods of depression and elevated mental state), suppressing menstruation and preventing jet lag. Bodies in capitalist economies have been imagined and exploited as cycling systems that resist increasing entropy, able to work like motors for as long as they can stave off and recover from fatigue as well as propagate the species (Rabinbach, 1992; Engels, 1972, pp. 25–26).

In these ways and more, life cycles and reproductive cycles have intersected with the other major cycles that have shaped human societies and their relations with the natural world, not least in work to extract resources. Talk of ‘genetic resources’ and ‘biocapital’ finds its motivation here and has its own images of circulation and flow (Bonneuil, 2019; Helmreich, 2008). Especially through the rise and fall of populations, and the Malthusian problematic of land, food and people, reproduction has been linked to economic cycles and to the transformations of matter.

## Transformations of matter

Great cycles of matter preoccupied much nineteenth-century science. Accounts of the perpetual motion of chemicals across the organic and inorganic worlds coupled life and death, agriculture and public health, generation and putrefaction in ways that highlighted the self-cleansing balance of nature—and minimized the dangers of pollution. Matter cycles played crucial roles in the politics of hunger and in chemists’ optimism about the power of their science to produce “an unbounded increase in organic life” (Justus Liebig quoted in Kamminga & Cunningham, 1995, p. 5). These cycles connected physiology, chemistry and civil engineering in the debates over sanitation and recycling, sewage and fertilizer, that wrestled with the crises of capitalist industrialization, themselves often framed as cycles. This is still relevant, because in the global circulation of the elements, as in the metabolism of nutrients within organisms, cyclic models of the transformation of matter retain their power.

During the eighteenth century, it had become standard for chemists to argue that matter circulated between living beings and inanimate nature (Teich, 1982). Buffon had “organic molecules” and other nutritive matters move through the biotic sphere (Bognon-Küss, 2019; Spary, 1996). Carl Linnaeus described “eternal circulation” as the order amidst the bustle of nature’s marketplace (Müller-Wille, 2003). Given the mounting acceptance that death and decay returned matter to the inorganic world, thus making it available for incorporation into new life, Linnaeus wondered if more use should not be made of the earth from cemeteries, enriched as this was by the cadavers of the just. But, he confessed, were “human heads” thus to be turned into “heads of cabbage”, he would have to be very hungry before he would eat them (quoted in Schramm, 1995, pp. 153–154).

The new chemistry, like the old, assumed the principle of the conservation of matter. Late eighteenth-century experiments showed that plants used carbon dioxide and produced oxygen for animals, whose respiration generated carbon dioxide again in a self-sustaining natural equilibrium. Justus Liebig’s agricultural chemistry posited cycles of nitrogen, phosphorus, carbon and potassium. Though confident in the capacity of the whole system to resist human disturbance, Liebig insisted that the loss of nutrients from the fields be made good. Also in the 1840s, the English social reformer Edwin Chadwick proposed a sewerage system to remove disease-causing human waste from the cities and use it to replenish the fields, while purifying the water and reducing the need to import guano as fertilizer. Old metaphors lived on when Chadwick justified this scheme as “complet[ing] the circle”, “by bringing as it were the serpent’s tail into the serpent’s mouth” (quoted in Schramm, 1995, p. 227). ‘Chemico-theologians’ invoked these principles when they touted the ‘balanced’ aquarium of 1850 as a demonstration of godly providence suitable for Christian parlours (Hamlin, 1986).

**Fig. 8:**
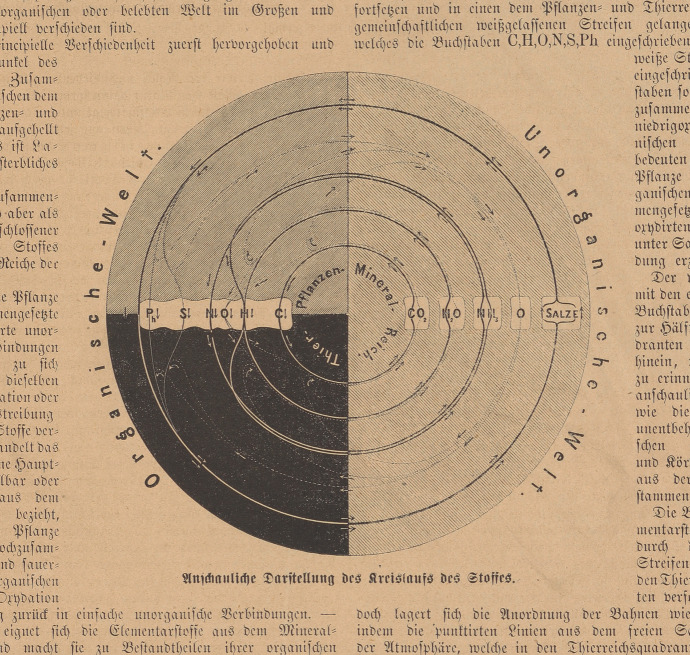
Graphical representation of the circulation of matter, designed to make vivid that, far from emptying the world of poetry, exact natural science had discovered in “the eternal circulation of matter” a “sober truth” that in its “overwhelming grandeur” and “fantastic charm” equalled the “highest and most sacred” myths (Czermak, 1872, pp. 356, 338). The circular area represents the universe, composed of the inorganic world or mineral kingdom (right) and the organic world (left), itself divided into plant kingdom (upper quadrant) and animal kingdom (lower). The arrows indicate how plants take up mineral salts, ammonia, water and carbon dioxide from the inorganic world by reduction reactions that produce complex compounds and release oxygen (dotted lines) into the atmosphere. The large white label representing those compounds straddles plant and animal kingdoms to indicate the reliance of animals on plants for nutrition. Oxidation in animal bodies (symbolized by reconnecting dotted lines) returns the salts, etc. to the mineral kingdom in their original forms. First displayed as a big diagram at a lecture by the physiologist Johann Nepomuk Czermak at his private amphitheatre in Leipzig in 1872, this version appeared as a wood engraving to accompany his essay in Germany’s leading family magazine. Czermak (1872, p. 355). Universitäts- und Landesbibliothek Darmstadt

Visions of universal circulation gained ground from mid-century. In the most successful synthesis, the materialist physiologist Jacob Moleschott drew on the ‘people’s scientist’ Georg Forster and the chemist Gerrit Mulder. Moleschott’s *Der Kreislauf des Lebens* (The circulation of life, 1852) and other books exalted the immortality of matter and the eternity of the cycle, driven now by matter itself, not God. “For just as trade is the soul of commerce, so the eternal circling of matter is the soul of the world” (Moleschott, 1857, p. 41). Secularizing Linnaeus, Moleschott marvelled at a process of exchange “that transforms our cadavers into the splendour of the fields and the flower of the field into the tool of thought” (Moleschott, 1853, p. 3). “For that is the noble creation of which we are daily witnesses, … that every individual being is only sacrificed for the species, that death itself is nothing but the immortality of the cycle [*Kreislaufs*]”. Wonder “lies in the eternity of matter through the change of form, in the change of matter from form to form, in metabolism [*Stoffwechsel*, literally ‘matter-exchange’] as primordial reason for life on earth” (Moleschott, 1857, pp. 86–87). Matter might be inexhaustible and immortal, but it did need proper distribution. The liberal Moleschott lamented that the Irish would never be strong enough to throw off the British yoke while they ate potatoes and not meat (Kamminga, 1995; Mårald, 2002; Meneghello, 2017; Schramm, 1995, pp. 211–222) (Fig. 8).

In 1995, Schramm’s history took aim at the quasi-religious faith in an almost alchemical purification through circulation in endlessly replenishing cycles, that boosted sewerage schemes while distracting from their practical failures to repair the destruction wrought by industrialization. In reality, the mining of minerals for fertilizer made the reuse of sewage unprofitable, so waste mostly ended up in the sea. Poisons accumulated and run-off nutrients caused eutrophication and other problems of their own. The recycling of human and other waste was still on the agenda in the twentieth century, but by then it was an uphill struggle to acknowledge the external costs (Bashford, 2014, pp. 189–194; Mårald, 2002; Schramm, 1995). For too long it had been enough to inveigh against the evils of stagnation and extol the blessings of circulation, in cities and hospitals as in aquaria (Vennen, 2018). Optimistic ontologies still guided the recycling of waste as animal feeds and nutrient media, and downplayed the toxicants that circulated as well (Landecker, 2019).

On a more specific level, nineteenth-century chemists recognized cycles of individual elements through the organisms that elaborated them into proteins, carbohydrates and fats, and so added content to the metaphor of a circulation or *Kreislauf* of organic matter. Liebig argued that animals did not only break down plant compounds; they also transformed them by building complex molecules from the simple ones they derived from food. The physiologist Claude Bernard underlined this indirect nutrition as a condition of organismal autonomy. He ignored the general terms for chemical changes in individual bodies, but ‘metabolism’ and Moleschott’s *Stoffwechsel* caught on (Bing, 1971; Landecker, 2017). Where Liebig presented all cycles as chemical, Louis Pasteur led germ theorists in pressing the claims of microorganisms as the agents of the decomposition that returned molecules to the earth. Biologically, even microscopic life arose not spontaneously, but from reproduction of the same species; chemically, the cycles connected the biotic and the abiotic as promiscuously as ever. Around 1900, research focused on the cycles of specific elements into and out of living things, and on the nutritional dependencies of various species. Sergei Winogradsky, a pioneer in the ecological microbiology of soil and water, investigated the roles of microbes in converting the multiple chemical forms of nitrogen (Ackert, 2013). In recent decades, the Winogradsky column, a glass cylinder filled with unsterilized mud, nutrients and water and exposed to sunlight, has become a much-used model of a balanced ecosystem in which elements can cycle between layers of different microbes for years (Grote, 2018).

Early to mid-twentieth-century biochemists, exploring the intricacies of metabolic processes within individual bodies, opened the ‘black box’ between digestion and excretion by analysing specific chemical cycles. The citric acid, or Krebs cycle, the sequence of aerobic reactions by which cells release stored energy as ATP, occupies the centre of the large charts of intermediary metabolism drawn by the Leeds microbiologist Donald Nicholson from 1955. Called after Hans Krebs, who discovered it in Sheffield in 1937, this became the paradigm of a whole set of metabolic cycles (Holmes, 1993, p. 429). But its designation as such was less obvious than is usually assumed and can serve as a further warning not to take talk about cycles too literally.

**Fig. 9:**
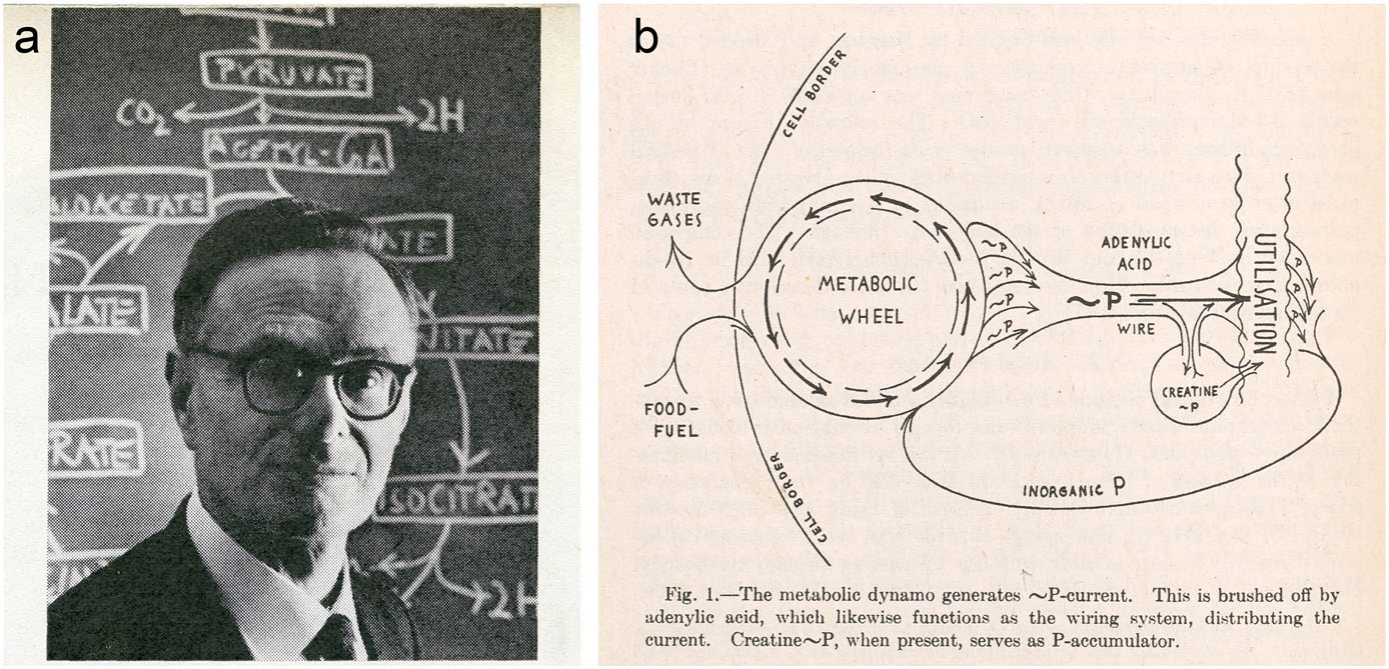
**a** Photograph of Hans Krebs in front of a blackboard showing the citric acid cycle, taken by Ted Polumbaum on 14 March 1963, possibly at Dartmouth College, for the *Life* magazine article “Nobel winners discover how metabolism works and what can go wrong” (1963, p. 64). Ted Polumbaum/Newseum collection. **b** Fritz Lipmann’s diagram of a “metabolic wheel” or “dynamo”. The “schematic representation” shows the “machine-like functioning of the revolving sequence of reactions”, with “brushed”, “wire” and “accumulator” evoking electric motors and circuits. Lipmann (1941, p. 122), © John Wiley and Sons

Krebs’s Berlin mentor Otto Warburg had already used the cycle motif for the reaction of a ferment (enzyme) and Krebs himself previously reconstructed the urea cycle. But Krebs told the historian of science Frederic Holmes that a diagram of that cycle was “merely a didactic, visual help”, that it was no different really from a series of equations that ended up back at the starting material (Holmes, 1991, pp. 429–431). Nor did Krebs then seek to generalize his urea work to what became the citric acid cycle; his scheme for those reactions was linear until late in the day (Holmes, 1993, pp. 399–401, 428–429) (Fig. 9a). But was there no essential difference between the generic ‘pathway’ and the specific form of a cycle? In a rare general discussion, Krebs pointed to the difficulty of telling irreversible and thus directional cycles apart from the simple oscillations of reversible reactions (Krebs, 1946). This might explain why the retrospective cycles of intermediary metabolism offer no metaphor as vivid as the “metabolic wheel” in Fritz Lipmann’s 1941 review of the generation and transfer of energy through phosphate bonds, those irreversible pacemaker reactions of cellular physiology (Fig. 9b).

Moleschott’s grand transformations of matter are ancestors of the ambiguous, esoteric cycles of modern biochemistry, in which enzymes are recycled as they catalyze diverse reactions. These still represent interlocking material conversions while also serving as mnemonic devices, reprinted countless times in textbooks and on posters to summarize the chemistry of life. Molecular biologists have explored living organisms as hierarchies of regulatory circuits; the physical chemist Manfred Eigen posited ‘hypercycles’ to explain the self-organization of replicating systems on the primordial Earth (Eigen & Schuster, 1979; Jacob, 1982). Moleschott’s transformations further inspired the ambitious cycles that ecologists would trace. But in the process his optimism about the natural ‘recycling’ of matter was tempered, given the dangers of environmental collapse that the metaphor effectively hides.

## Ecological cycles, systems and feedback

From biochemistry to ecology and demography to geology, much of the twentieth-century and especially the postwar history of the sciences of population and the environment can be framed in terms of two kinds of cycles and the relations between them: biogeochemical cycles of the flow of elements and compounds in units as small as a pond and as big as the planet; and population cycles, or periodic fluctuations in the number of individuals in a species. The latter were linked substantively to the former, the main type of cycle in ecology, through the reliance of organisms on food and hence on the nutrients, principally fixable nitrogen, that fed the fertility of the soil and water, though this hardly exhausts the content of population studies (Bashford, 2014). Biogeochemistry and biodemography were connected formally through their regulation by negative feedback. Thanks to multiplying connections and a swelling repertoire of relations, simple circle imagery expanded into complex representations of systems and circuits while retaining the vocabulary of cycles and circulation. In the process, closed cycles were opened up.

**Fig. 10:**
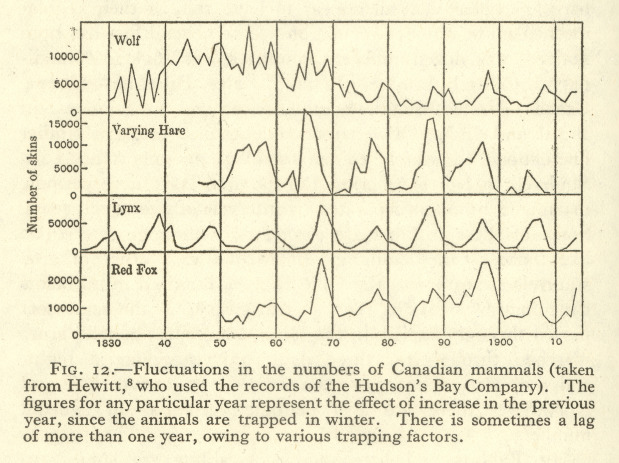
Graphs of population cycles of Canadian mammals (numbers of skins per year) in Charles Elton’s textbook. Elton directed attention to the “valuable fur-bearing” wolf, lynx and red fox, “which subsist” “on the varying hare or snowshoe rabbit (*Lepus americanus*)”. Elton (1927, p. 136)

Population ecology came out of the application in the 1920s of statistical physics to interactions between predators and prey. The solutions to the coupled differential equations of the mathematicians Alfred Lotka and Vito Volterra “trace out sinusoids in time, with predators and prey chasing each other in never-ending cycles of feast and famine, … boom and bust” (Erickson, 2010, pp. 533–534; Hagen, 1992; Kingsland, 1985). Mixing linear, cyclic and network metaphors, the Oxford zoologist Charles Elton influentially advocated the approach in a textbook that set the problem of population cycles in the framework of food chains. “[A]ll the food-chains in a community” formed the “food-cycle”, he explained, or what caught on as the “food web”, while behaviour was modified by diurnal rhythms, tides, the seasons and climatic change (Elton, 1927, p. 56). Elton had learned about the periodic fluctuations of Arctic mammals on an Oxford University expedition to Spitsbergen and as a consultant to the Hudson’s Bay Company. Ecology promised to help smooth market fluctuations in the exploitation of fish, feathers and fur. Mathematical analysis of historic trapping records let him process the results into something approaching regular cycles (Fig. 10). In 1930, at a Conference on Biological Cycles in Matamek, Newfoundland, a gathering of Canadian wildlife experts and academics, Elton met the Yale University geographer Ellsworth Huntington. Huntington tried to explain business cycles in terms of the effects of sunspots, through climate, on economic activity. Elton adopted that climatic determinism, but was forced by contradictory evidence to abandon it in the 1940s. By the 1950s, mathematicians and ecologists were debating the size and regularity of the periodicity that could count as cyclic; aspects of population cycles remain mysterious even today (Anker, 2001; Erickson, 2010; Huntington, 1931; Jones, 2017).

Elton had included infectious disease as a special kind of density-dependent predation, and its potential as a structuring force became a theme of ‘disease ecology’, the combination of microbiology and animal ecology that flourished in Anglo settler societies dealing with new environments (Anderson, 2004). In these studies of the coevolution of host-parasite interactions and their (sometimes cyclic) effects on population, the U.S. veterinary pathologist Theobald Smith’s stress on interacting life cycles tended to be lost in favour of linear life histories. The growing emphasis on microevolutionary processes fitted well with the older idea that competition drives progress towards modernity—and, it seemed, the regular decline of parasite (or microbe) virulence and rise of host resistance. In mathematical models promoted from the 1970s this was relegated to just one of an array of options, none explicitly cyclic, to explain disease emergence and spread (Anderson, 2016; Jones, 2017; Méthot, 2012). In the twenty-first century, however, the new field of planetary health, the study of the effects of climate change and global environmental degradation on human health, has increasingly referred to circular processes and feedback loops (Dunk & Anderson, 2020).

**Fig. 11:**
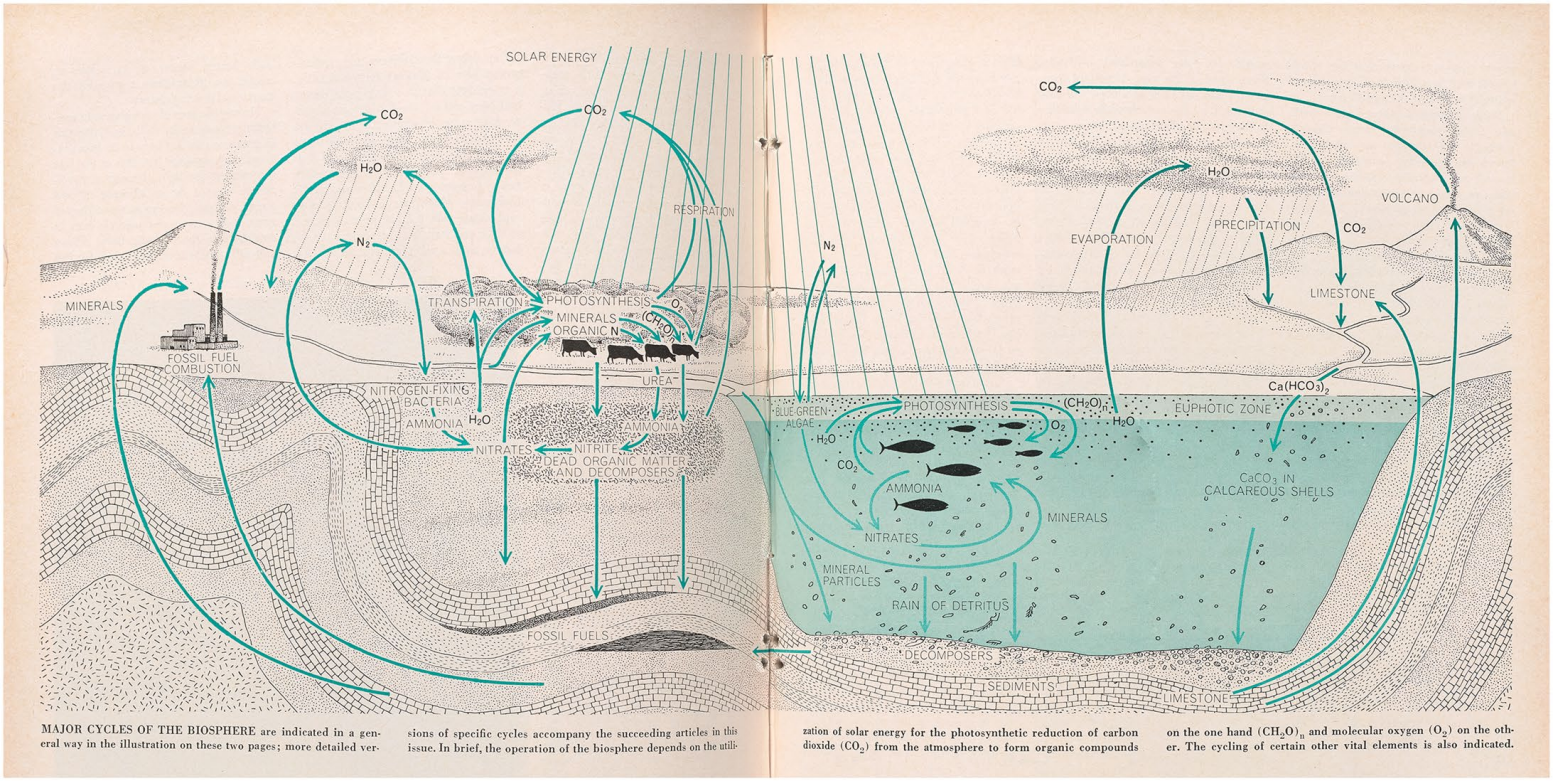
Diagram of major cycles of the biosphere by the prolific illustrator Bunji Tagawa, from Evelyn Hutchinson’s introduction to an influential *Scientific American* issue on the topic. Compare Fig. 8, this article. Hutchinson (1970, pp. 50–51). Courtesy of the estate of Bunji Tagawa

Cycles provided more obvious organizing metaphors in biogeochemistry than in biodemography and epidemiology. Researchers into lakes and other waterways traced nutrient flows between living organisms and inorganic reservoirs—water, air and soil—and started to diagram these as cycles in the mid-1920s. The approach was generalized in the 1940s by G. Evelyn Hutchinson, the British professor of zoology at Yale, who drew on the Russian mineralogist Vladimir Vernadsky’s vision—venturing quantitative estimates for the fluxes of matter into and out of the biosphere—to elevate his own studies of carbon and phosphorus cycles in a local lake into a global model. Hutchinson’s student Raymond Lindeman gave this research programme momentum by reinterpreting and mathematicizing Elton’s trophic dynamics, while distinguishing cycling matter from the energy that drove those cycles with its one-way flow. This cleared the path to unify studies of producers, consumers and decomposers in a whole set of element-specific cycles through the living and non-living compartments of ‘ecosystems’. It gave systemic content to Cambridge botanist Arthur Tansley’s concept (Hagen, 1992, p. 90) (Fig. 11).

World War II and the Cold War shaped ecosystem cycles as these prevailed in the United States. Hutchinson presented an expansive account of “circular causal systems in ecology” in 1946 at one of the Macy Conferences that advanced cybernetics. Coming fresh out of research on missile-guidance mechanisms, cyberneticians touted “circular systems” as a key to “self-regulating processes, self-orientating systems and organisms, and self-directing personalities” and made “feedback” a hallmark of cyclicity (Frank, 1948, p. 191; Hutchinson, 1948). Hutchinson linked biogeochemical and biodemographic modes of ecological analysis by their responsiveness to negative feedback. In the carbon cycle the main mechanisms were a reversible chemical equilibrium and the irreversible photosynthetic cycle. Against growing concern that burning fossil fuels was raising the concentration of carbon dioxide in the atmosphere—he suggested deforestation as a more likely cause—Hutchinson pointed to the ability of the vast ocean reservoir to buffer the system. He described the sigmoid curves of populations as increasing to a saturation level, the ‘carrying capacity’; and argued that this testified to the power of feedback, as did the limitation by natural selection of the amplitude of oscillations in the numbers of predators and prey (Hagen, 1992; Hutchinson, 1948). Cybernetics provided a language for the deep commitment to equilibrium that Hutchinson shared with many in the postwar United States, and ‘negative feedback’ expanded the appeal of thinking in cycles. But as in biochemistry with the idea of regulation, where the product inhibits its own over-production, that thinking often formalized notions that were current already but had gone by other names (Creager & Gaudillière, 1996, pp. 49–52; Holmes, 1995).

Hutchinson had gestured to the tight links between ecology and biochemistry by writing in 1940 of the “metabolism” of a community, even as he, like Tansley, rejected the assumption that an ecosystem behaved like an organism. The Atomic Energy Commission funded ecologists and biochemists to refine cycles by radioisotope tracing and they produced similar diagrams of nutrient cycles and metabolic pathways, for example, the Calvin-Benson cycle in photosynthesis (Creager, 2013; Nickelsen, 2015).

**Fig. 12:**
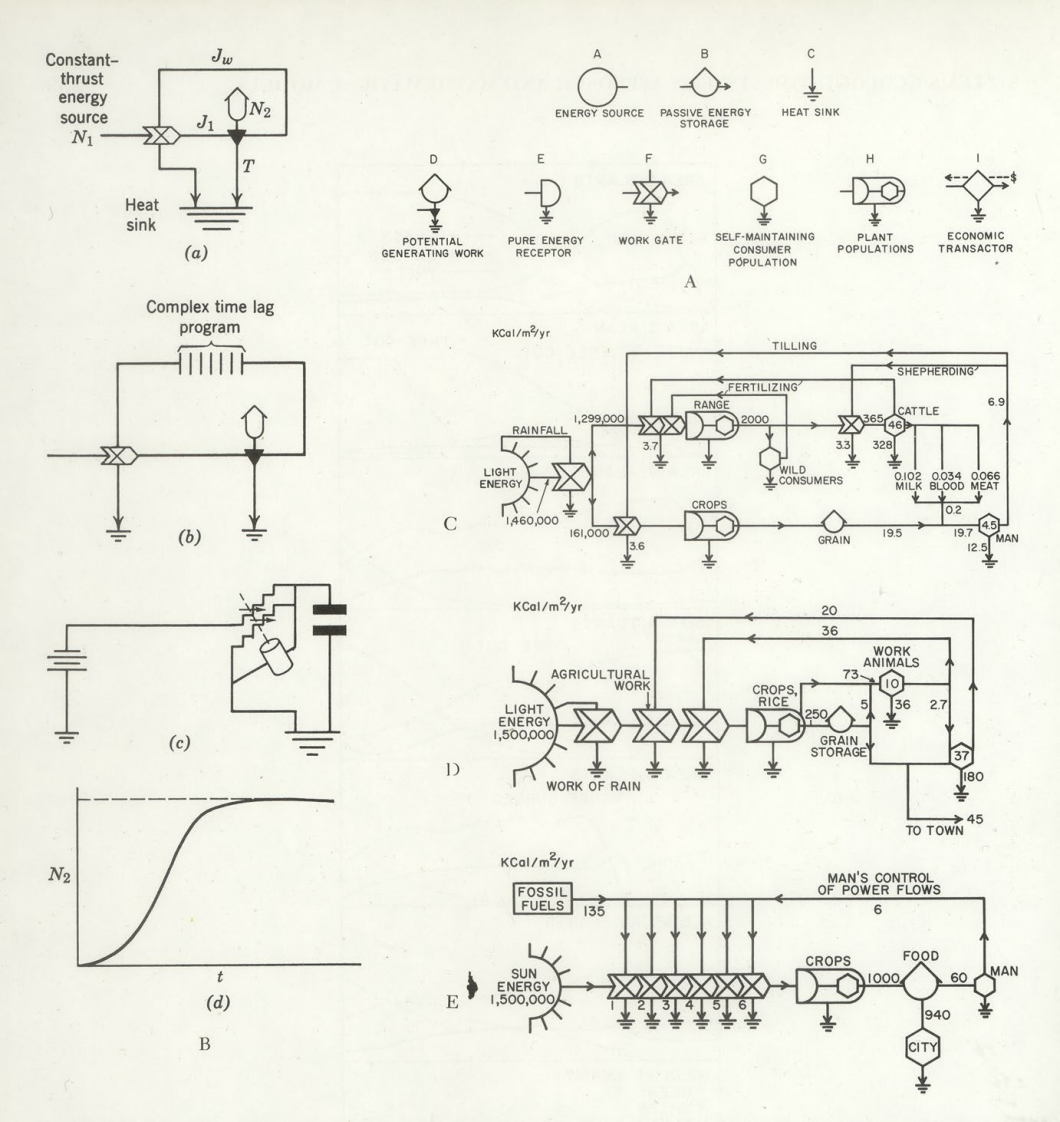
“Examples of electrical analog circuit models” after Howard Odum in a chapter by Carl J. Walters in Eugene Odum’s textbook. **A** Energy network symbols, **B** circuit diagrams and graph of logistic growth to carrying capacity, and three energy circuits that “form an evolutionary series” from **C** a tribal cattle system in Uganda, through **D** unsubsidized monsoon agriculture in India, to **E** the fuel-subsidized industrial agriculture of the United States. The text emphasized the need for “models that consider the country and the city as one integrated and mutualistic life-support system”, unlike the common, but “extremely dangerous and misleading” practice of “sub-optimizing” a model for food production only without considering environmental stress or economic and social consequences. Reprinted from Odum (1971, p. 290) with permission from Elsevier

Cycles played an ever more general role in the U.S. ecosystem ecology broadcast by the brothers Eugene and Howard T. Odum, but as researchers abandoned faith in equilibrium, and in the power of the global system to self-regulate, closed cycles gave way to open ones (Kingsland, 2005, pp. 199–221). Howard Odum developed an energy-circuit notation which, though idiosyncratic, spread through his brother’s market-leading textbook. Howard Odum used these circuits to debunk the myth that U.S. agriculture achieved high yields through sunlight and hybrid seeds alone; that ignored the input from fossil-fuel-based fertilizers, pesticides, feedlots and air-conditioning (Fig. 12). Neither academic ecologists nor their funders took much notice of this argument—but organic farmers and environmentalists did. Rachel Carson’s *Silent spring* (1962) gained power from showing how poisons entered the “endless cyclic transfer of materials from life to life” and stopped the “wheel” from “turning” in nature’s energy-producing “cycles of renewal” (Carson, 2000, pp. 56, 179; Madison, 1997). The ideal of a return to an original state, itself often understood as a healthy cycle, today underpins much conservation and restoration work (Martin, 2015).

Born of worries about world population, environmentalism further connected population cycles and biogeochemical cycles. The nitrogen cycle, in particular, linked land (also water), food and people, and neo-Malthusians pushed waste recycling alongside contraception to heal what, in an influential book, the eugenicist and conservationist Henry Fairfield Osborn Jr called *Our plundered planet* (1948). Becoming prominent around 1970, environmental activism sought to prevent the disaster of numbers of people expanding exponentially until they exceeded the carrying capacity of the Earth. An influential model of boom and bust held that the population would then crash as the species ran out of food or perished from disease, the whole process perhaps to be repeated in a dystopian cycle (Bashford, 2014). Some environmentalists, seeking to escape overcrowding here, succumbed to the lure of the Space Age, and made the circulation of materials in a cabin a futurist model for colonies on other planets. The prototype of a self-contained environment, Biosphere 2, was built in Arizona between 1987 and 1991 to show how Earth (‘Biosphere 1’) could be redesigned more rationally (Anker, 2005; Aronowsky, 2017).

Yet not only was population control increasingly discredited as imperialist, the reproduction that it aimed to halt has also come to seem more precarious since the 1970s, not least through the effects of toxic environments on, for example, sperm counts. Even in sub-Saharan Africa, where population is growing rapidly today, untreated infertility is a major concern alongside high rates of neonatal and maternal death. Modern fears of population decline go back to eighteenth-century France but, as Natasha Zaretsky has written, “the ecological age is marked above all by a new awareness that reproduction’s predictive logic—its circles and cycles, its recurrences and repetitions, its rhythms and rhymes, its eternal returns—no longer applies to our world” (Zaretsky, 2018, p. 201). That vision may be overly dark, but in our present predicament it is difficult to recover the optimism of metaphors of restoration, let alone progress. Cycles, however, remain at the heart of understanding human impacts on Earth and of attempting to mitigate them.

## Conclusion

The theme of cycles and circulation, at the centre of human hopes for personal reproduction and fears for planetary survival, offers a fresh look at the history of medicine and biology over the long term. The cyclic and circulatory metaphors we have surveyed differ widely—some mere oscillations, others highly structured—and have had complex relations with other geometrical figures. Many stories can and should be told about them, but some patterns are clear. Cycles are apt to share the purpose of reconciling the unity, individuality and endurance of life with the insight that it also progresses, dissipates and expires. Highlighting this general tendency alerts us to the specific powers of cyclic representations to make certain relationships and processes visible while obscuring others.

Above all, we have explored relations and distinctions between metaphors of cycles and circles. Whether static or in motion, circles symbolize perfection and individuality. Self-contained, they have no beginning or end, and so facilitate the communication of dynamism and flow. Once emancipated from circles, cycles have had varied shapes as well as defined points of return, but their users have still tended to favour circular forms. Representations in the shape of a circle may be topologically equivalent to other geometric figures, such as the square; lines of equations may express the same thing as a circular diagram; a series of pictures can include every stage of a life cycle—and yet, a cycle of stages represented in circular form, especially with added arrows, gives a stronger image of return. That is true even if it hides what may in fact be a linear progression. These modern cycles are less closed, more contingent and hence more open to human intervention; they more pointedly convey movement towards some endpoint or product. This ambivalence between closed and open, and relations to circles that have ranged from the cosmological to the molecular, give the cycle metaphor a diverse and complex history.

As representations of processes, cycles have not just competed, but also intersected with figures of linear change, the ladders, lines and trees of progress or decline. The finite, directional histories of the Abrahamic religions opposed the general assumption of endless cycles. Geology and evolutionism subsequently underpinned the ideologies of progress that cyclic models were sometimes used to block or soften, but evolutionary theorizing itself posited change through cycles of development and reproduction. Alternatively, Christian theologians may be seen as having posited a single cycle of paradise, fall and resurrection that was challenged by the Darwinist insistence on radical contingency—now as the result of natural law not the work of an active God.

The *Encyclopaedia Britannica* once presented the “tendency to undergo cyclical changes” as a distinguishing feature of living matter (Thiselton-Dyer, 1878, p. 679). Yet if life cycles perpetuate species and delimit organisms, cycles of matter pose the problem of maintaining life in the face of constant molecular exchange with the inorganic world. Cycles have been too widely used, not least in astronomy and for machines, ever really to have set life apart. Crossing boundaries and with them vast differences of scale, the metaphor of cycles has mediated exchange among the life sciences and, for example, with economics and geology. If life is not uniquely cyclic, the association of women and periodic or cyclic change has been durable and influential; the gendering of cycles invites further research.

In our own profession of history of science, cycles have organized accounts of intellectual change. Kuhnian revolutions implied a cycle of crisis and resolution, and since they went out of fashion, circulation has become another pervasive metaphor. James Secord suggested foregrounding investigations into the conditions by which knowledge circulates, and this approach has much to recommend it still (Secord, 2004). Yet the apparently innocuous term hides long-buried meanings, including of the heart as a pump and the vessels as infrastructure, greater awareness of which would promote more appropriate usage. If these older resonances have aided the appeal of *circulation* within global history by construing the globe as a closed system, that field has also embraced the open flows and exchanges that the word implies (Gänger, 2017). But just as in economics circulation rarely leads to an equitable exchange, so postcolonial historians have argued that the metaphor risks masking asymmetries of power and conflicts in the production of knowledge (Anderson, 2009; Fan, 2012; Raj, 2013, p. 344).

More generally, while circles seem to impose an orderly return, no single politics or ideology has monopolized notions of cycles. Conservatives have mobilized them against progressive and revolutionary ideas, and presented political and economic crises as merely another turn of the wheel. But cyclic thinking can be enlisted to break down barriers, as in Moleschott’s insistence on free trade in interconvertible matter. Marxists traditionally emphasized production at the expense of circulation, but historical materialism posits the cyclic resolution of crises through revolutions. Political meanings are never far away when cycles are invoked.

Since the early nineteenth century, idealizations of cyclic movements have tended to provide reassurance about progress, representing steady states or stadial developments while facilitating change and the exploitation of resources for industrialization. Cycles have been harnessed for their reproductive power and suppressed where they interfered with productivity. The rise of an ecological consciousness depended, by contrast, on identifying threats to natural cycles of life. Following the dominance in postwar ecology of closed, ahistorical cycles, the acknowledgment of all kinds of inputs and outputs, gains and losses, has modified any illusion of stability. Environmentalists have shown that it is artificial to represent industrial systems without recognizing these additional cycles. In this way, they have stressed how much is at stake in conceptualizing one cycle rather than another and thus projecting different futures. Like circles, cycles may be either vicious or virtuous—or both.
